# Healing of tendon-related diseases: insights from macrophage regulation

**DOI:** 10.1186/s40779-025-00635-x

**Published:** 2025-08-04

**Authors:** Ren-Qiang Chen, Peng-Ju Liu, Shuai Li, Hong-Pu He, Dan-Mei Li, Guang-Xun Yuan, Xiang-Yu Du, Jing-Yue Su, Zhen-Han Deng, Jian Xu

**Affiliations:** 1 Department of Orthopedics, the First Affiliated Hospital, Zhejiang University School of Medicine, Hangzhou, 310003 Zhejiang China; 2 Department of Orthopedics, the Fourth Affiliated Hospital of Guangxi Medical University, Liuzhou, 545005 Guangxi China; 3 Department of Orthopedics, the First Affiliated Hospital of Wenzhou Medical University, Wenzhou, 325000 Zhejiang China; 4 Geriatrics Center, the First Affiliated Hospital of Wenzhou Medical University, Wenzhou, 325000 Zhejiang China

**Keywords:** Tendon, Tendon-bone interface (TBI), Tendon-related diseases (TRDs), Macrophages, Tissue engineering

## Abstract

Tendon-related diseases (TRDs) are increasingly common in the current aging society and impose a significant burden on patients. Despite therapeutic advances, the pathophysiology of TRDs remains poorly understood, hindering effective clinical management. The macrophages are highly plastic immune cells involved in the maintenance of in vivo homeostasis and the injury-healing process. Their dual role in TRDs has been widely investigated, either promoting tenogenic and chondrogenic differentiation or amplifying inflammatory response, underscoring their therapeutic potential for TRDs treatment. Therefore, the review aims to summarize the roles of macrophages in the healing of TRDs, characterized by limited regenerative capacity, and examine strategies for the modulation of macrophage phenotypes to accelerate the regeneration process. Finally, we review applications involving macrophage modulation within the context of tissue engineering of TRDs, providing novel insights for the design of biomaterials-based targeted delivery systems.

## Background

Tendons serve as critical components within the musculoskeletal system, connecting bones to muscles and facilitating the transmission of muscular forces [1]. The high incidence of tendon-related diseases (TRDs) has been attributed to the essential and frequent use of tendons. A previous study has reported approximately 1.71 billion musculoskeletal injuries annually worldwide, with half attributed to TRDs [2]. In the general population, the prevalence of rotator cuff tears (RCTs) is estimated to be 20.7%, with a higher prevalence with increasing age [3]. Tendinopathy is estimated to cost approximately $850 billion annually, imposing a significant economic burden on society [4]. Currently, treatments for tendon or tendon-bone interface (TBI) healing include conservative and surgical management. Each approach presents distinct advantages and disadvantages. For instance, oral nonsteroidal anti-inflammatory drugs are associated with adverse effects such as gastrointestinal ulcers and hepatorenal damage. Even after surgical intervention, the natural hierarchical structure of the TBI often fails to regenerate, being replaced by fibrous scar tissue [5]. The mechanical properties of scar tissues are compromised, which makes them prone to rupture under normal stress and leads to high recurrence rates [5, 6]. Therefore, the effective repair of tendon and TBI injuries remains a significant concern in clinical settings.

Macrophages, key innate immune cells distributed throughout the circulatory system and peripheral tissues, play a critical role in maintaining tissue homeostasis [7]. They respond to pathogens and senescent cells by initiating physiological responses to maintain health [8]. The polarization process of macrophages allows them to possess diverse functions through the modulation of their phenotypes [9]. Consequently, subtypes like M1 and M2 macrophages exhibit unique functions when subjected to various stimuli. M1 macrophages promote inflammation, whereas M2 macrophages drive tissue repair and proliferation. Imbalances in macrophage subtypes are increasingly recognized as a key etiology in various musculoskeletal diseases, including osteoarthritis [10], Duchenne muscular dystrophy [11], and TRDs [3]. Macrophage-mediated unresolved inflammation in TRDs leads to excessive fibrous tissue formation, peritendinous adhesions, and suboptimal tendon healing. The polarization of macrophages toward the M2 phenotype and reducing M1 macrophage abundance have proven effective in addressing these challenges [12, 13]. In the field of tissue engineering (TE), techniques that direct macrophage polarization for TRDs management have gained significant momentum. Recent studies have shown that the combination of scaffolds, mesenchymal stem cells (MSCs), and cytokines can promote tendon regeneration by enhancing M2 polarization and suppressing the M1 phenotype [14–16]. Scaffold parameters, bioactive factors, and drugs play an essential role in macrophage modulation. Thus, targeting molecular mechanisms of macrophage polarization and modulating macrophage balance through TE provides a promising therapeutic strategy for TRDs.

However, a comprehensive synthesis of macrophage polarization in the context of TRDs repair applications, including its cellular mechanisms and integration with TE, remains limited. Consequently, this review aimed to summarize key examples of macrophage polarization in both tendon and TBI healing processes. We then explored the repair mechanisms involving potential signaling pathways and evaluated the progress in enhancing polarization. Finally, we presented validated prospective models and highlighted strategies for future consideration to accelerate regeneration of tendon and TBI through macrophage polarization.

## Overview of macrophages and their interaction with MSCs

**Fig. 1:**
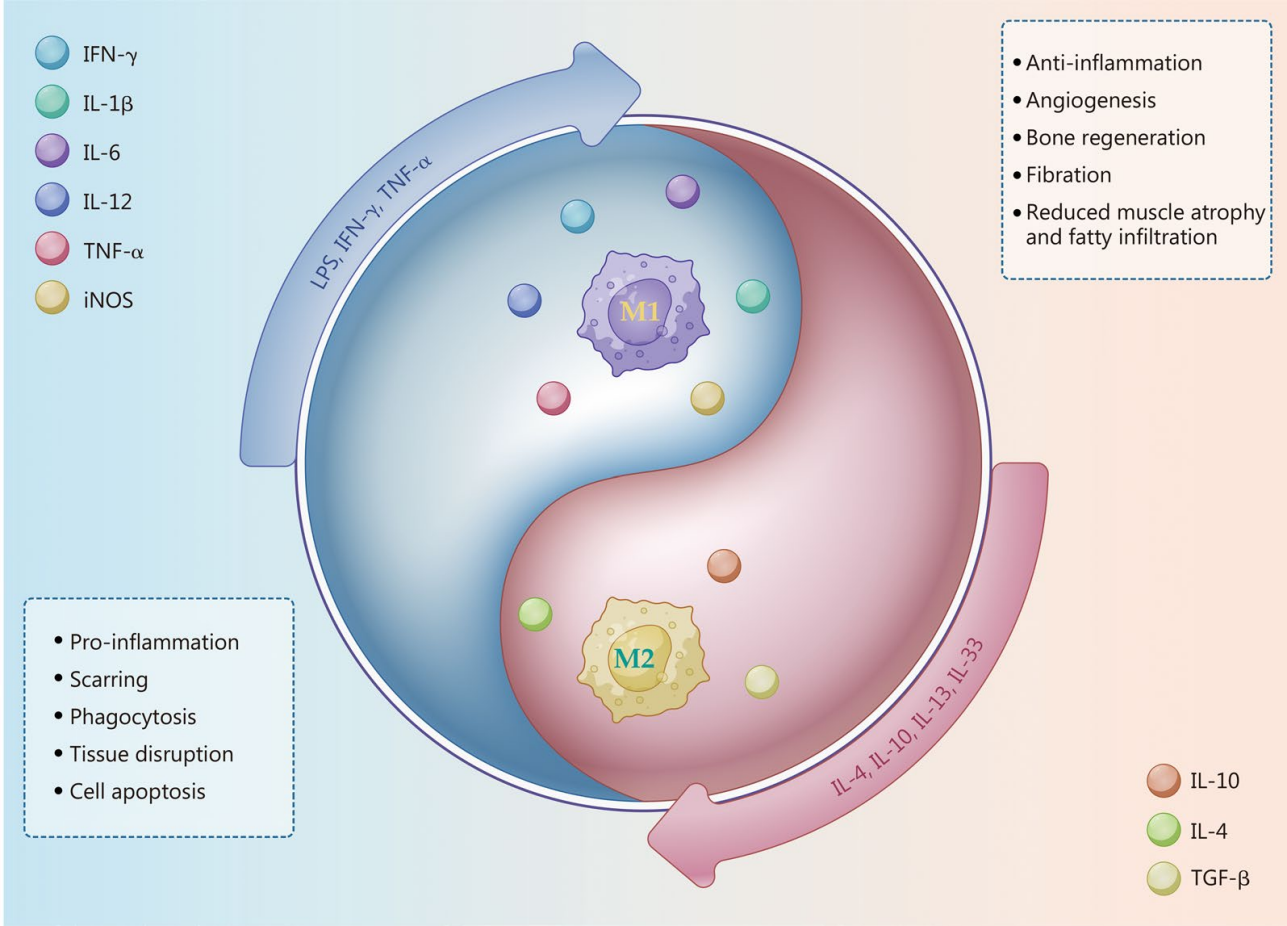
An overview of macrophages. Activated by stimuli such as LPS, IFN-γ, and TNF-α, M1 macrophages secrete pro-inflammatory cytokines (e.g., IL-1β, IL-6 and IL-12), which are linked to pro-inflammatory responses, scarring, phagocytic activity, tissue disruption, and cell apoptosis; while M2 macrophages are activated by cytokines like IL-4, IL-10, IL-13, and IL-33, promoting anti-inflammatory effects, angiogenesis, bone regeneration, fibrillation, and reducing muscle atrophy and fatty infiltration. M1 pro-inflammatory macrophages, M2 anti-inflammatory macrophages, IFN-γ interferon-γ, IL interleukin, TGF-β transforming growth factor-β, TNF-α tumor necrosis factor-α, iNOS inducible nitric oxide synthase, LPS lipopolysaccharide

First identified by Metchnikoff [17] in the nineteenth century, macrophages, as key innate immune cells, have been extensively studied over the past century. Macrophages are now understood to be widely distributed across all body tissues, playing critical roles in both physiological and pathological processes through their diverse functions, such as phagocytosis, homeostasis maintenance, and immune responses to inflammation [9, 18]. For decades, the prevailing understanding was that tissue macrophages originate solely from circulating monocyte differentiation; however, a study has demonstrated that yolk sac progenitor cells are also a significant source [19]. Tissue macrophages integrate environmental signals to orchestrate processes such as tissue growth, remodeling, and homeostasis [20]. Depending on the signals received, activated macrophages are primarily categorized into M1 and M2 phenotypes, each with distinct characteristics and functions (Fig. 1). This review focused on the M1 and M2 phenotypes, exploring their activation mechanisms, gene expression profiles, roles in tissue repair, and interactions with MSCs.

### M1 macrophages

Resting macrophages (M0) undergo polarization into the M1 phenotype upon activation by T helper type I cytokines, such as tumor necrosis factor-α (TNF-α), interferon-γ (IFN-γ), or microbial lipopolysaccharide (LPS), leading to overexpression of cluster of differentiation 80 (CD80) and CD86 [19, 21, 22]. The IFN and Toll-like receptor (TLR) signaling pathways activate the classical interferon regulatory factors/signal transducer and activator of transcription (IRF/STAT) pathways, shifting macrophage toward the M1 phenotype [23]. As key mediators of inflammation, M1 macrophages secrete high levels of pro-inflammatory chemokines, including TNF-α, interleukin-1β (IL-1β), IL-6, IL-12, and IFN-γ [3]. These chemokines not only propagate inflammatory responses but also modulate the activity of surrounding cells. Elevated levels of IL-1β and TNF-α have been shown to suppress the tenogenic activity of MSCs [24] and enhance bone resorption by increasing osteoclast activity [25]. Besides, M1 macrophages exhibit lower expression of IL-10, a cytokine critical for tissue regeneration, compared to M2 macrophages [26]. Therefore, while M1 macrophages function as scavengers of bacteria and apoptotic cells, they may also compromise surrounding healthy tissues, contributing to extracellular matrix (ECM) degradation. Indeed, macrophages significantly influence ECM turnover by secreting proteases and the aforementioned chemokines. They also modulate fibroblast collagen production and autonomously synthesize matrix metalloproteases (MMP) and tissue inhibitors of matrix metalloproteases (TIMP) [27]. Co-culture with M1 macrophages upregulates the activity of collagen-degrading proteases in tendon fibroblasts, including MMP2, MMP3, and MMP9 [28]. Furthermore, M1 macrophages mediate tissue damage via excessive production of reactive oxygen species (ROS) [19]. It has been established that LPS-induced M1 activation leads to ROS accumulation in macrophages, impeding the transition to the M2 subtype and hindering tissue regeneration [29]. Metabolically, M1 macrophages exhibit enhanced anaerobic glycolysis to meet the energy demands for rapid pro-inflammatory molecule synthesis [30].

### M2 macrophages

M2 macrophages are typically activated by Th2 cytokines, including IL-4, IL-10, IL-13, and IL-33, and are characterized by high expression of CD163, CD206, and arginase-1 (Arg-1) [21, 22]. They play a pivotal role in tissue regeneration and remodeling. Specifically, transforming growth factor-β (TGF-β), IL-4, IL-10, and growth factors enhance the activity of fibroblasts and endothelial cells, promoting collagen production and angiogenesis [31]. However, M2 macrophages may contribute to pathological fibrosis or scar tissue formation [32, 33], due to the role of TGF-β in collagen synthesis [34]. Moreover, M2 macrophages can be further classified into M2a, M2b, M2c, and M2d subtypes based on signals from the surrounding microenvironment [3]. M2a macrophages, activated by IL-4 and IL-13, promote tissue healing by expressing CD206, growth factors (TGF-β, insulin-like growth factor), and decoy IL-1 receptors [7]. M2b macrophages are considered regulatory macrophages, as they secrete both anti-inflammatory cytokines (e.g., IL-10) and pro-inflammatory cytokines (e.g., IL-1β, IL-6, and TNF-α) [7]. M2c macrophages, induced by IL-10 and glucocorticoids, function as anti-inflammatory macrophages by secreting IL-10, promoting fibroblast proliferation via TGF-β, and phagocytosing dead cells via Mer receptor tyrosine kinase [3, 7]. M2d macrophages, recently identified as tumor-associated macrophages [3, 35], are induced by TLR ligands or IL-6 and secrete IL-10 and vascular endothelial growth factor (VEGF), promoting tumor vessel formation and metastasis. Notably, the subtypes and functional plasticity of macrophages cannot be fully captured by the M1 and M2 phenotypes alone. However, these phenotypes represent the two extremes of the macrophage activation spectrum, which most scholars consider key polarization directions warranting further exploration [36].

### The interaction with MSCs

MSCs exhibit multilineage differentiation potential, enabling tenogenesis, chondrogenesis, and osteogenesis [3, 37]. MSCs residing in tendons are referred to as tendon-derived stem cells (TDSCs), distinguished from other MSCs by their higher expression of tendon-related genes [38]. MSCs contribute to the healing of TRDs not only due to their multilineage potential but also through their immunomodulatory functions. Upon detection of damage-associated molecular patterns (DAMPs) via TLRs, MSCs secrete pro-inflammatory factors such as C–C motif chemokine ligand 5, C-X-C motif chemokine ligand 9, and macrophage inflammatory protein-1 to recruit macrophages to the injury site [39]. It has been shown that microRNA-223 (miR-223) in MSC-derived exosomes promotes M2 macrophage differentiation and cutaneous wound healing [40]. Studies have explored modulating macrophage phenotypes using MSCs pretreated with pro-inflammatory cytokines [41] or melatonin [42]. Notably, TDSCs in chronic or aged RCTs exhibit a senescent phenotype, promoting M1 polarization, which in turn exacerbates TDSCs senescence and creates a positive feedback loop [5]. Given this, although the study has highlighted the beneficial role of MSCs in promoting M2 polarization, their effectiveness remains constrained by the functional status of MSCs. Moreover, macrophage-mediated inflammation impairs the tenogenic differentiation capacity of TDSCs [43]. Pro-inflammatory cytokines secreted by M1 macrophages not only inhibit MSC osteogenic and chondrogenic differentiation but also enhance osteoclast activity [6]. Kang et al. [44] demonstrated that exosomes from M2 macrophages could enhance osteogenic differentiation by upregulating bone morphogenetic protein (BMP)-2 and BMP-9. Similarly, Li et al. [45] found that M2 macrophages secrete TGF-β1 to recruit MSCs, which differentiate into myofibroblasts and promote peritendinous adhesion. To accelerate the healing of TRDs, the complex interactions between MSCs and macrophages should be modulated through tissue engineering strategies.

Taken together, macrophages play essential roles in both inflammation and tissue repair in vivo through paracrine effects on the surrounding tissues and MSCs. Macrophages exhibit different phenotypes (e.g., M1, M2a, M2b) through polarization depending on tissue conditions. However, categorizing macrophages into these subtypes is overly simplistic, as polarization is a dynamic process, and current research still cannot confirm these phenotypes as terminally differentiated. Macrophages should instead be viewed as a dynamic continuum, capable of reversible state transitions upon stimulation. However, the majority of studies on macrophages in TRDs still use the basic M1/M2 classification. Future research needs to go beyond this simplification, particularly by integrating the functional states of subcellular organelles.

## Natural healing of tendons and TBI

### Natural healing of tendons

**Fig. 2:**
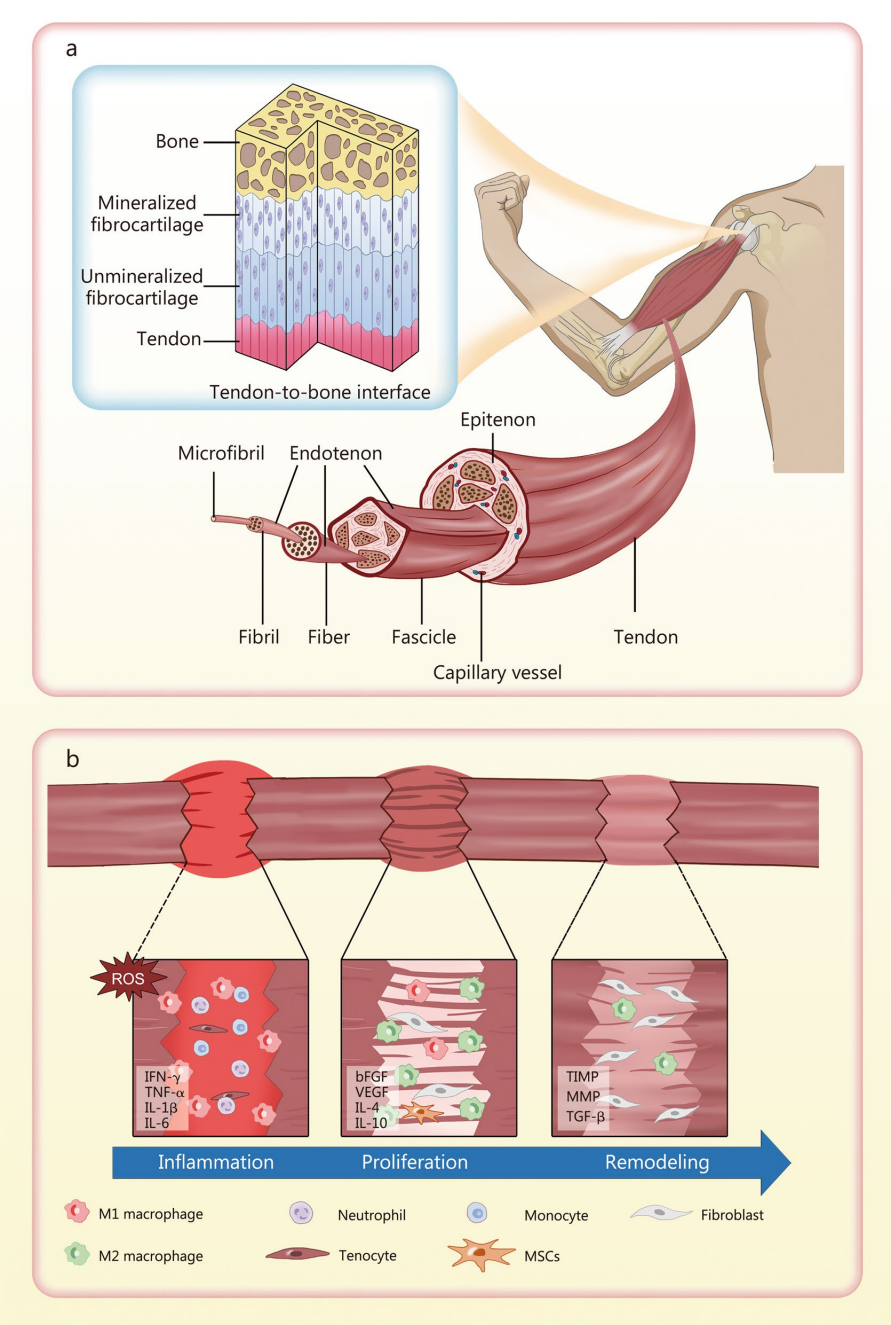
Schematic illustration of the structure and healing process of TRDs. **a** The structure of the tendon and the tendon-to-bone interface. **b** The sequential healing process of the tendon. M1 pro-inflammatory macrophages, M2 anti-inflammatory macrophages, ROS reactive oxygen species, IFN-γ interferon-γ, TNF-α tumor necrosis factor-α, IL interleukin, bFGF basic fibroblast growth factor, VEGF vascular endothelial growth factor, TIMP tissue inhibitors of matrix metalloproteases, MMP metalloproteases, TGF-β transforming growth factor-β, MSCs mesenchymal stem cells

The native structure of tendons is hierarchically organized through the sequential aggregation of microfibrils (10 nm), fibrils (50–500 nm), fibers (10–50 µm), fascicles (50–300 µm), and tendons (1–10 mm). Histological analysis has established that the tendon ECM is primarily composed of type I collagen (Col I), synthesized by resident tenocytes [46]. Following acute injury, tendon regeneration is a naturally occurring, well-orchestrated process involving 3 sequential phases of inflammation, proliferation, and remodeling [31, 47] (Fig. 2). The acute inflammation phase following tendon injury initiates the repair process through the release of numerous immunological cells and cytokines, including IFN-γ, TNF-α, IL-1β, and IL-6 [48, 49]. Recruited macrophages and monocytes in this phase also engulf the bacteria and cell debris. During the proliferation phase, pro-regenerative M2 macrophages increasingly secrete growth factors such as VEGF, IL-4, IL-10, and fibroblast growth factor, which promote angiogenesis and collagen synthesis [3, 31, 48, 49]. The fibroblasts exhibit enhanced synthetic activity, leading to the deposition of type III collagen (Col III) at injury sites [29], rather than the tendon’s primary component, Col I [50]. In the subsequent remodeling phase, cell density and synthetic activity decrease, while the newly synthesized ECM undergoes more pronounced realignment [49]. While Col I may replace Col III in the remodeling phase [29], the natural tendon structure is often incompletely reconstructed, resulting in healed tendons with only one-third of the tensile strength of healthy tissue, thereby increasing the risk of retear [48]. Moreover, the tendon healing process is divided into intrinsic and extrinsic processes. Intrinsic healing is characterized by tenocyte proliferation and ECM synthesis, whereas extrinsic healing is driven by fibroblast infiltration from adjacent tissues [51]. Due to the limited number of tenocytes and the relatively low activity of growth factors, the extrinsic pathway predominantly influences tendon repair, often resulting in scar tissue formation.

### Natural healing of TBI

The TBI, also known as enthesis, connects soft tendon/ligament tissues to hard bone, preventing stress concentration during body movement [6]. It is commonly involved in RCT pathology and ligament reconstruction procedures, such as anterior cruciate ligament repair. The TBI structure is complex due to its role in force dissipation and consists of 4 distinct zones: tendon, unmineralized fibrocartilage, mineralized fibrocartilage, and bone [6] (Fig. 2). Although these 4 zones are continuous and interconnected, they exhibit distinct components [52]. The tendon zone comprises aligned Col I fibers and fibroblasts [53]. The unmineralized and mineralized fibrocartilage zones are dominated by unmineralized and mineralized fibro-chondrocytes, respectively, with less organized collagen. The bone zone contains osteoblasts, osteocytes, and osteoclasts and comprises mineralized Col I. TBI healing shares similar characteristics with tendon healing, including inflammation, proliferation, and remodeling [3]. Notably, the efficiency of tendon-bone healing is positively correlated with the degree of bony ingrowth and TBI tissue maturation [54]. However, once damaged, the anatomical layered structure of TBI is difficult to restore and is often replaced by fibrovascular scar tissue [55]. Scar tissue consists of disorganized ECM rather than aligned collagen fibers, leading to poor integration with bone and weaker mechanical properties [56]. Moreover, the poor vascularization of the TBI further limits its intrinsic healing potential and impedes bone ingrowth [3]. Consequently, balancing the inflammatory response and promoting synchronous regeneration are imperative for achieving optimal TBI healing.

## Macrophages in the healing of TRDs

**Fig. 3:**
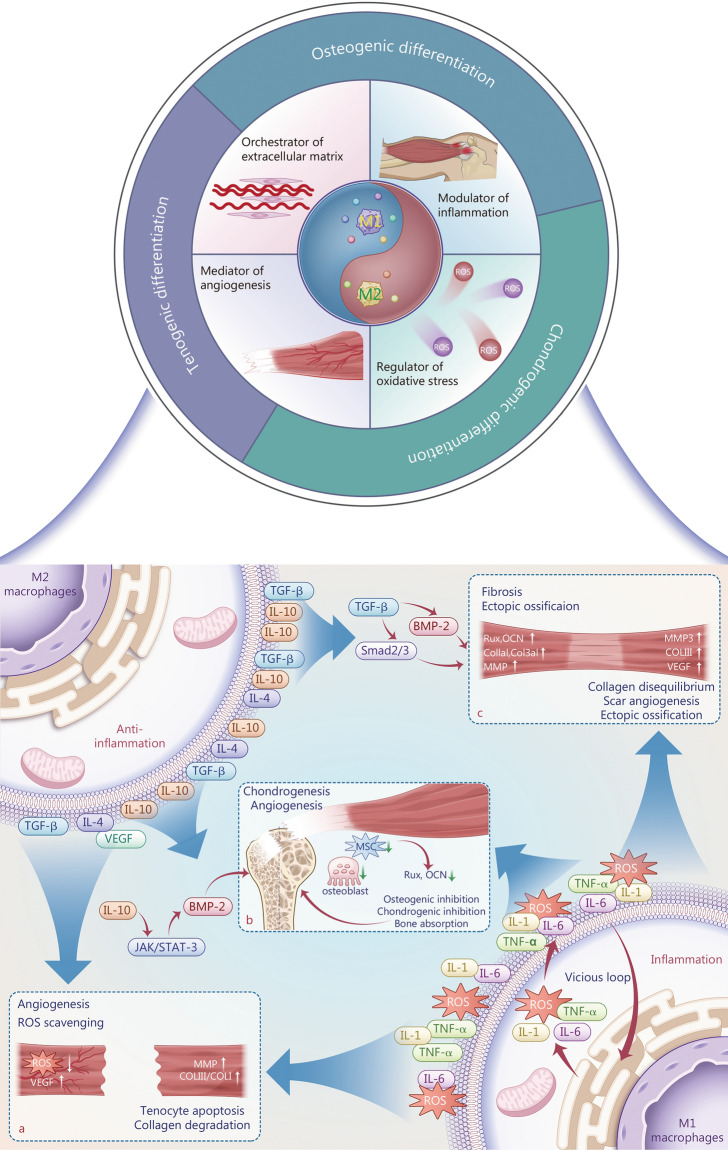
An overview of the influence of macrophages in the healing of tendon-related diseases. **a** Macrophage in the healing of tendon rupture. **b** Macrophage in the healing of tendon-to-bone interface. **c** Macrophage in the healing of tendinopathy. M1 pro-inflammatory macrophages, M2 anti-inflammatory macrophages, TGF-β transforming growth factor-β, IL interleukin, VEGF vascular endothelial growth factor, BMP-2 bone morphogenetic protein-2, JAK/STAT Janus kinase/signal transducer and activator of transcription, ROS reactive oxygen species, MMP metalloproteases, Col I type I collagen, Col III type III collagen, MSCs mesenchymal stem cells, Rux ruxolitinib, OCN osteocalcin, COI 1a1 type 1a1 collagen, COI 3a1 type 3a1 collagen, TNF-α tumor necrosis factor-α

As previously discussed, the post-injury healing process of tendons and TBI comprises 3 continuous stages. Throughout these stages, macrophages, owing to their functional diversity, play a complex role in tissue repair by modulating inflammation, oxidative stress, angiogenesis, fibrosis, and eventual ECM remodeling (Fig. 3). The success of healing depends on the precise coordination of each stage. Insufficient inflammation may lead to inadequate clearance of necrotic tissue and bacteria, whereas an excessively prolonged healing process can result in fibrotic scar formation.

### Modulator of inflammation and immune microenvironment

Following tissue injury, DAMPs are released and trigger inflammation, recruiting neutrophils and monocytes to injury sites, which are later dominated by macrophages. In addition to a small proportion of tissue-resident macrophages in the musculoskeletal system, there is a robust influx of extrinsic macrophages migrating to injury sites [57]. M1 macrophage abundance peaks on the first day post-injury and returns to baseline after two weeks, whereas M2 macrophages tend to increase after 28 d [33]. Specifically, following tendon rupture, the acute hematoma that contains numerous cytokines and chemokines, recruits classically activated M1 macrophages to phagocytose necrotic cell debris [49]. M1 macrophages infiltrate tendon injury sites and secrete pro-inflammatory cytokines which are detrimental to tissue repair, such as IL-1β, IL-6, and IFN-γ [14]. By sustaining inflammation, these cytokines exacerbate tissue damage and disrupt regenerative mechanisms, potentially delaying the healing process. They can both inhibit the regeneration of the TBI fibrocartilage layer and enhance osteoclast activity [6]. In later stages, to repair injury sites, M1 macrophages have to polarize into the M2 phenotype, which secretes anti-inflammatory cytokines (e.g., IL-4, IL-10, and IL-13) and growth factors [34], promoting the resolution of inflammation. Overall, M1 and M2 macrophages secrete cytokines and chemokines that collectively regulate the immune microenvironment in TRDs. The M2 macrophage-dominated immune microenvironment promotes tenogenic expression in tenocytes and enhances osteogenic signaling, thereby facilitating tendon and TBI healing [6, 58]. Nevertheless, M1 macrophages also release chemokines such as C–C motif ligand 2 (CCL2) and stromal cell-derived factor-1 to recruit MSCs for early repair [59], indicating their beneficial role despite their pro-inflammatory functions.

### Regulator of oxidative stress

There exists a strong association between oxidative stress and inflammation. Given the growing body of research on modulating macrophage polarization to reduce ROS production in tendon regeneration [29, 43, 60], it is necessary to discuss oxidative stress as a section. Following tendon injury, ROS accumulates during macrophage polarization. Excessive ROS damages the mitochondrial enzyme system of tenocytes, reduces adenosine triphosphate (ATP) synthesis, and inhibits TDSCs proliferation and tenogenic differentiation [43, 60]. Moreover, excessive ROS production can drive M1 polarization and increase pro-inflammatory mediators, creating a vicious loop between the imbalanced immune microenvironment and tendon injury [60]. Li et al. [61] demonstrated that ROS induces cell cycle checkpoint activation, leading to cell cycle stagnation and subsequent M1 macrophage activation. In contrast, M2 macrophages exhibit an anti-inflammatory phenotype, contributing to the resolution of oxidative stress. Thus, promoting M2 macrophage polarization can reverse these effects and establish a regenerative microenvironment conducive to TRDs repair. Accordingly, strategies to suppress ROS production, such as combining ROS-responsive materials [62], antioxidant nanozymes [29], and antioxidant traditional Chinese medicines [43], are currently and will remain a research focus in TRDs therapy.

### Mediator of angiogenesis

Tendons exhibit high metabolic activity and substantial nutritional demands, underscoring the critical role of vascularization [55]. However, tendons are hypovascular tissues, posing a significant challenge to the healing of TRDs. M2 macrophages, characterized by a pro-angiogenic phenotype, promote endothelial cell angiogenesis by secretion of VEGF, TGF-β, and basic fibroblast growth factor [31, 63]. Although M1 macrophages also stimulate angiogenesis, direct cell-to-cell contact between these macrophages and endothelial cells inhibits tube formation in vitro [64]. The inflammatory microenvironment induced by M1 macrophages is detrimental to angiogenesis in the early stages of TBI injury [65]. Subsequently, transitioning from M1 to M2 macrophages promotes angiogenesis and facilitates tissue repair. However, the role of angiogenesis in specific TRDs remains inconsistent. In acute tendon and TBI injuries, neovessel formation enhances collagen deposition and osteogenic differentiation [31, 65]. Growing evidence indicates that the histological features of tendinopathy are characterized by pathological angiogenesis [15, 66]. Pathological angiogenesis in tendinopathy could disrupt normal tendon structure through the deposition of Col III [15]. Huang et al. [67] demonstrated that the blood supply surrounding the TBI, rather than vascular ingrowth, promotes TBI healing. While M2 macrophage polarization promotes angiogenesis, pathological vascularization can lead to tendons losing their normal structure and promoting the formation of fibrovascular scar tissue. Consequently, modulating macrophages for angiogenesis depends on the specific context. Treatment for tendinopathy should focus on inhibiting M2 macrophage polarization to suppress pathological vascularization [15], whereas early tendon and TBI healing require stimulating M2 macrophages [31].

### Orchestrator of fibrosis and ECM remodeling

Macrophages not only participate in inflammation and cellular debris phagocytosis but also regulate ECM synthesis and degradation. The balance of MMP and TIMP secreted by macrophages is crucial for tendon ECM turnover. Compared to acute tendon injury, MMP-2 and MMP-13 are upregulated in tendinopathy, leading to increased expression of type III and type V collagen [68]. Macrophages exhibit differential expression of these enzymes. First, M1 macrophages secrete pro-inflammatory cytokines linked to matrix degradation [28], and in regions dominated by M1 macrophages, the expression of MMP-1 and MMP-13 is elevated [69]. Second, M2 macrophages, characterized by a pro-healing phenotype, promote ECM homeostasis [28]. However, a study has also shown that M2 macrophages promote fibrosis and peritendinous adhesion formation by secreting TGF-β1, which recruits MSCs and induces their differentiation into fibroblasts [45]. Thus, excessive activation of M2 macrophages may lead to fibrotic repair rather than natural tendon regeneration. This suggests that an appropriate M1/M2 ratio is essential for promoting ECM remodeling at all stages of tendon repair. Indeed, further investigation is needed into the crosstalk between macrophages and MSCs or fibroblasts. For example, TDSCs, the primary MSCs in tendons, are divided into subgroups with distinct differentiation potentials, including tenogenic and fibrogenic progenitors [70]. Whether M1 and M2 macrophages regulate the homeostasis of the tendon ECM through crosstalk with these two types of TDSCs remains unclear. Meizlish et al. [71] recently demonstrated that macrophages could sense the mechanical properties of ECM synthesized by fibroblasts and negatively modulate ECM synthesis to prevent fibrosis under physiological conditions, offering new insights for suppressing adhesive tissues in TRDs.

## Strategies to promote macrophage polarization in the healing of tendon and TBI

### Mechanical stimulation (MS)

MS plays a critical role in the repair of TRDs. Controlled mechanical loading of healing tendons enhances cell infiltration and proliferation, induces collagen deposition and promotes tenogenic differentiation [72, 73]. Macrophages play an essential role in these processes. Many characteristics of biomaterials, including stiffness, roughness, and wettability, are associated with mechanical stimuli, making them a research hotspot in recent years. Therefore, we devote a section to MS to illustrate its role in influencing macrophage polarization for TRDs treatment.

#### The range of mechanical stimulation for regulating macrophage polarization

Macrophages are mechanosensitive and modulate their polarization in response to different mechanical cues. Wang et al. [52] demonstrated that MS could promote macrophage polarization toward the M2 phenotype and enhance MSC chondrogenesis in a TBI model. Macrophages subjected to mechanical stretch typically exhibit an anti-inflammatory phenotype [74]. And macrophages are more responsive to physical cues than tendon fibroblasts [75], indicating the important role of macrophages in mediating physical stimulation to promote tendon repair. Notably, excessive mechanical loading can prolong tendon healing [32], while immobilization promotes TBI healing by increasing M2 macrophage abundance and eliminating M1 macrophages [76], highlighting the importance of appropriate MS for macrophages. The research found that low strain levels (5% or 7%) could induce M2 polarization, whereas high strain levels (12% or 15%) could induce M1 polarization [74, 77]. However, macrophages cultured on substrates with random topography exhibited pro-inflammatory characteristics when exposed to similar strain levels (7%) [75]. The differences in these studies cannot be entirely attributed to MS parameters. The source of macrophages also determines their functional properties, indicating that bone marrow-derived macrophages and tissue-resident macrophages may exhibit different phenotypes even under the same kind of MS. Research focusing on macrophage mechano-responsiveness should note such differences and explain with caution when comparing with the previous literature. Moreover, musculoskeletal tissues are primarily subjected to uniaxial and biaxial stretching rather than vascular shear stress [78], which may explain the distinct responsiveness of macrophages.

#### Approach for the application of mechanical stimulation for macrophage polarization

Various approaches are currently available to apply MS to macrophages, ranging from engineered biomaterial stiffness [79] and topography [79] to treadmills in animal experiments [80], and combining with other stimuli (e.g., ultrasound and electromagnetic fields) [81]. While these approaches can achieve mechano-regulation of macrophages, they present some notable limitations. For instance, firstly, biomaterials may trigger immune rejection, and their degradation products can alter macrophage behavior [82]; secondly, mechanical loading devices (e.g., the Flexcell Tension System) often have complex setups and apply asymmetric mechanical loads [83], limiting their ability to precisely modulate cellular activities. Thus, designing a stable and effective microenvironment for MS remains a significant challenge. Emerging micro/nanorobot technologies have been used to generate mechanical signals via magnetic or electric fields. With the assistance of magnetic fields (MFs), self-assembled microrobots can rotate around macrophages, generating mechanical signals that block the Piezo1 mechanosensitive activation pathway and promote M2 polarization [83]. This approach achieves immune regulation in a simple, controllable, and non-invasive manner. Furthermore, magnetic nanoparticles, with their excellent tissue penetration, can serve as mediators to introduce oscillations in MFs. These oscillations function as a form of MS and induce macrophage differentiation. Research has shown that high-frequency oscillations promote M1 polarization, whereas low-frequency oscillations promote the M2 phenotype [84]. Future mechanical stimulation systems should achieve real-time adaptation to dynamic cellular mechanobiological demands while minimizing damage to the surrounding tissues.

#### Form of mechanical stimulation for macrophage polarization

In vivo, both blood and tissue macrophages are exposed to dynamic MS, including hydrostatic pressure and cyclic stretch induced by blood pressure [78]. These differential mechanical inputs elicit distinct macrophage responses. Zhang et al. [85] revealed that cyclic compression could upregulate M2 markers and bone regeneration markers while simultaneously downregulating M1 markers. Ballotta et al. [77] previously assessed the effects of different cyclic strain levels (0%, 7%, and 12%) on phenotype polarization and matrix deposition, finding that 7% cyclic strain could promote reparative macrophage polarization and ECM synthesis. In contrast, static strain has been reported to enhance the expression of inflammatory macrophage markers [78]. However, Atcha et al. [86] demonstrated that both cyclic and static loading could inhibit IFN-γ/LPS-related inflammation. While it is evident that dynamic MS critically regulates macrophage behavior in vivo, current evidence does not yet establish clear guidelines for the precise MS parameters (mode, magnitude, frequency) required to direct macrophage differentiation, highlighting key directions for future research. Besides, cyclic stretch reduces macrophage degradative activity toward biomaterials, whereas static stretch increases it [87], offering valuable insights into the design of biomaterials for the management of TRDs.

### Biomaterials

TE has emerged as a promising strategy to promote the regeneration of both tendons and the TBI [6, 16]. Upon transplantation into the body, biomaterials trigger immune responses that either establish a pro-regenerative microenvironment or induce chronic inflammation for tissue development. Macrophages, as innate immune cells, are undoubtedly crucial regulators of this process. Therefore, it is essential to investigate how biomaterials modulate macrophage behavior and optimize biomaterial parameters to enhance outcomes in TRDs treatment. Key biomaterial parameters, including stiffness, surface topography, roughness, and biochemical factors, are discussed below.

#### Biomaterial classification for the modulation of macrophage polarization

Biomaterials used to modulate macrophage polarization are primarily categorized into natural and synthetic biopolymers. This section summarizes the biomaterial factors influencing M2 macrophage polarization and their advantages in healing TRDs (Table 1) [88–106]. Briefly, natural biomaterials, due to their superior biocompatibility, tend to induce M2 polarization, whereas synthetic biomaterials, owing to their immunogenicity, are more likely to trigger pro-inflammatory responses. However, both natural and synthetic materials can be engineered to enhance their immunoregulatory properties (Table 2) [63, 107–115]. Adhikari et al. [108] revealed that the incorporation of magnesium (Mg) particles into a polycaprolactone (PCL) nanofiber scaffold could reduce inflammatory responses compared to PCL alone. Osteoporosis presents a significant challenge in TBI healing, particularly in aging populations. Niu et al. [115] found that acetylated *Bletilla striata* could accelerate M1 polarization and promote the production of a range of pro-osteogenic cytokines. To reverse osteoporotic bone metabolism, Wu et al. [112] developed a citrate-functionalized scaffold that inhibits metabolic enzyme activity in M1 macrophages, thereby promoting M2 polarization. More importantly, citrate degradation products function as anti-inflammatory agents to promote bone healing [112]. Methods for functionalizing biomaterials include free-radical copolymerization [114], diazonium-based chemistry [116], and electrospinning [108]. The functionalized biomaterial coatings, by modulating tissue macrophages to improve host-scaffold integration, show potential in addressing the immune imbalance in TRDs.

**Table 1:** The biomaterials applied in the induction of M2 macrophage polarization for TRDs

Biomaterials	Factors affecting M2 polarization	Strengths for TRD healing	References
Chitosan	Degrees of acetylation, molecular weight	Biocompatibility, anti-peritendinous adhesion	[88–90]
Gelatin	RGD polypeptide, hydrophilic	Biodegradability, sustained release	[91, 92]
Hyaluronic acid	Molecular weight, hydrophilicity	Enhancing TDSC’s synthesis of type I collagen	[93–95]
Decellularized ECM	Dimension of the hydrogel (2D and 3D)	Promoting TDSC’s differentiation	[96, 97]
Alginate	Ease of modification, structural similarity with ECM	Robust mechanical properties, biodegradability	[98, 99]
PLGA	3D printed structure mimicking ECM	Biodegradability, biocompatibility	[100, 101]
PLLA	Hydrophobicity, aligned topography	Biocompatibility, controllable biodegradability	[95, 102, 105]
PCL	Nontoxic degradation, aligned topography, weak hydrophilicity	Slow degradation, strong mechanical strength	[103, 104, 106]

*RGD* arginine-glycine-aspartic acid, *ECM* extracellular matrix, *PLGA* poly lactic-co-glycolic acid, *PLLA* poly-L-lactic acid, *PCL* polycaprolactone, *TDSC* tendon-derived stem cells, *2D* two-dimensional, *3D* three-dimensional

**Table 2:** The functional agents used in the modification of biomaterial to modulate macrophages

Functional agents	Effects on macrophage	Examples	References
Organic			
Acetylation groups	Accelerated M1 polarization	*Bletilla striata* polysaccharide	[115]
Carboxyl groups	Impaired the expression of M2 scavenger receptor	Polystyrene-COOH	[114]
Amino groups	Decreased the phagocytosis ability of both M1 and M2 macrophages	Polystyrene-NH_2_	[114]
Amide groups	Reduce the M1 macrophages and increase M2 polarization	Poly (N-isopropylacrylamide-co-acrylic acid)	[113]
Citrate groups	Inhibit the metabolic enzyme activity of M1 macrophages	CPC@PCL/CaCit	[112]
Glycosylated groups	Restoring M2 macrophage polarization after LPS stimulation	Glycosylated nano-hydroxyapatites	[111]
Sulfated groups	Reduce expression of the pro-inflammatory cytokine of M1 macrophages	Sulfated alginate	[110]
Inorganic			
Silver nanoparticles	Reduce the M1 macrophages and increase M2 polarization	Gallic acid-silver nanoparticle composites	[109]
Magnesium	Stimulating M2 polarization and decreasing surrounding inflammation	Magnesium-polycaprolactone nanofibers	[108]
Copper	Downregulating M1 and upregulating M2 marker expression	Copper-incorporated bioactive glass–ceramics	[107]
Strontium	Promoting M2 polarization	Strontium-doped mesoporous bioglass nanoparticles	[63]

*M1* pro-inflammatory macrophages, *M2* anti-inflammatory macrophages*, LPS* lipopolysaccharide

Moreover, synthetic polymers exhibit superior mechanical properties to natural polymers, which are often too weak to withstand the stresses associated with tendon movement [75]. The mechanical properties of natural polymers can be improved by using composite polymers that combine synthetic and natural polymers. Nevertheless, Fu et al. [117] recently developed an all-natural hydrogel hybridized with protocatechuic aldehyde and collagen, demonstrating its superior properties in immune modulation and angiogenesis. Compared to synthetic polymers, readily prepared all-natural polymers can reduce the recruitment of pro-inflammatory macrophages and form a pro-regenerative microenvironment, indicating new insights for the application of immunoregulatory biomaterials.

#### Stiffness

Biomaterial stiffness is a critical regulator of cellular functions, morphology, and migration. Macrophages not only alter their functional phenotypes but also interact with local cells via paracrine signaling upon activation by material stiffness [24]. The polarization trend of macrophages is closely related to substrate stiffness. Macrophages exposed to soft substrates were found to secrete increased levels of IL-1β and ROS compared to those on stiff substrates, thereby exacerbating the inflammatory microenvironment of injured tendons and reducing tenogenic expression [24]. Chen et al. [118] reported that low material stiffness could promote the polarization of bone marrow-derived macrophages toward the M1 subtype. However, other studies have contrastingly revealed that soft substrates could promote M2 polarization, whereas stiff substrates could promote M1 polarization [79, 119, 120]. These discrepancies may arise from various factors, including distinct cell sources and culture dimensions. Friedemann et al. [121] compared the activation profiles of macrophages cultured in three-dimensional (3D) and two-dimensional (2D) collagen matrices. Macrophages in the 3D matrices expressed higher levels of pro-resolving cytokines, while those in 2D matrices showed increased secretion of both pro-resolving and pro-inflammatory cytokines.

Piezo1 is a category of mechanosensitive calcium channels, responding to the matrix stiffness [120]. Upon Piezo1 activation, it regulates macrophage polarization via the mechanotransduction effector molecule Yes-associated protein (YAP). A stiff matrix promotes YAP expression and nuclear localization, thereby enhancing M1 polarization. In contrast, YAP inhibition boosts M2 polarization, offering a new target for designing immunomodulatory biomaterials [120]. The stiffness property reflects the mechanical properties of materials [122], and is influenced by material porosity, pore size, and the interconnectivity between pores [123]. Therefore, when designing material stiffness to modulate macrophage-mediated repair of TRDs, the impact on other parameters must be taken into consideration. The cryoprotectants applied during gelatin scaffold fabrication could decouple the control of both pore structure and stiffness [124]. This offers a solution for precisely regulating pore size without affecting other parameters.

#### Roughness

Scaffold roughness is a critical factor in cell-surface interactions. An optimal roughness enhances cell adhesion and spreading by influencing protein adsorption. However, the effect of roughness on macrophage polarization remains controversial. While increased substrate roughness has been documented to elevate inflammatory cytokine expression in macrophages [125, 126]. Barth et al. [127] reported that macrophages on rough surfaces exhibit secretory profiles similar to M2 macrophages, due to the following reasons: 1) the hydrophilicity may impact the effects of roughness; 2) the ranges of roughness applied in different studies vary. Generally, hydrophobic biomaterials activate pro-inflammatory macrophages, while hydrophilic surfaces suppress this process. Notably, hydrophilic rough titanium surfaces promote increased levels of anti-inflammatory cytokines IL-4 and IL-10 [128], highlighting the superior immunomodulatory impact of surface wettability compared to surface roughness.

Recent advances in lithography techniques have refined material roughness to micrometer and nanometer scales, allowing for a more comprehensive understanding of surface topography. Zhang et al. [129] found that only a narrow range of micrometer-scale roughness could promote M2 macrophage polarization. Dabare et al. [130] fabricated nano-rough substrates with different scales (16, 38, and 68 nm) by immobilizing gold nanoparticles on a 2-methyl-2-oxazoline thin film. Macrophages on all modified surfaces exhibited an anti-inflammatory phenotype and downregulated the inflammatory gene expression. Thus, discrepancies in the impact of roughness on immune regulation may arise from roughness parameters, material wettability, and surface chemical modifications, all of which should be considered in designing material roughness. The Wnt signaling pathway is reportedly involved in macrophage interactions with material roughness [131], and inhibiting canonical Wnt signaling could reduce pro-inflammatory macrophages and cytokines, regardless of surface roughness or wettability [132]. As a key macromolecule for mechanical sensing in macrophages, Piezo1 regulates macrophage polarization and inflammatory pathways by integrating diverse mechanosensory signals [122]. However, it remains to be determined whether an association exists between Wnt signaling and the Piezo1 molecule in the context of TRDs healing.

#### Topography

Upon implantation within the in vivo environment, the topography of biomaterials can either trigger host immunological rejection or promote healing with surrounding tissues. An ideal substrate topography not only reduces pro-inflammatory secretion but also facilitates tissue regeneration and anti-inflammatory responses. Topography influences gene transcription by altering cell shape and function upon adhesion to implants [133]. Consequently, previous studies have explored modulating macrophage phenotypic changes by modifying the topography [134, 135]. McWhorter et al. [135] demonstrated that aligned topography induced a pro-healing macrophage phenotype mediated by actin and myosin contractility. In contrast, disorganized biomaterial topography was found to trigger the release of inflammatory cytokines from macrophages [75]. Notably, scaffolds with aligned morphology have been shown to enhance tenogenic differentiation, suggesting a dual benefit when using aligned designs in biomaterials [136].

Electrospinning, lithography, and other microfabrication techniques enable precise control of substrate topography and help elucidate macrophage modulation mechanisms. Luu et al. [134] designed titanium surfaces with micro- and nanopatterned grooves using deep etching techniques. Their results demonstrated that surface grooves enhanced macrophage elongation, with an optimal width of 400–500 nm which correlated with elevated M2 macrophage activation. Monteiro et al. [137] created biomimetic topographies using soft lithography and found that fibroblasts or ECM-mimetic surfaces induced M2 phenotypic expression, whereas macrophages cultured on pathogen-mimetic topographies exhibited an M1 phenotype. However, these 2D cultured systems often fail to fully represent the in vivo environment, despite their excellent controllability. In contrast, 3D topography provides a microenvironment that mimics physiological conditions and offers a greater surface area for cell adhesion and proliferation. Jiang et al. [138] developed expanded 3D nanofiber scaffolds with higher porosity, which enhanced macrophage infiltration and achieved a higher M2/M1 ratio after subcutaneous implantation in rats. Tendons are primarily composed of Col I, which exhibits highly aligned topography. Ryma et al. [139] first introduced melt electrofibrillation to fabricate collagen-mimetic nanofibril bundles, demonstrating that biomimetic 3D topography mimicking native Col I promoted M2-like polarization, comparable to standard IL-4 stimulation. Furthermore, co-culturing MSCs with macrophages in a 3D topographical system significantly reduced inflammatory secretion, including IL-6 and monocyte chemotactic protein-1, compared to 2D topography [140]. Although 3D topographical patterns are challenging to fabricate [141], they provide a biomimetic microenvironment that supports macrophage polarization and tenogenic expression, facilitating the healing of TRDs.

### Targeted delivery

While the aforementioned MS and biomaterial properties are beneficial, achieving precise modulation of macrophages remains a significant challenge. The specific inflammation-associated proteins on macrophage surfaces are relatively conserved [142]. Consequently, targeted delivery treatments, such as those using ligands to deplete pro-inflammatory macrophages or inhibit their gene expression, have emerged as feasible strategies, including drugs, genes, and bioactive components. Specifically, several key mechanisms have been identified by which these delivery treatments promote macrophage polarization toward the M2 phenotype (Table 3) [5, 14, 31, 43, 47, 65, 80, 143–148]. In this section, we review strategies for delivering drugs, genes, and exosomes to modulate macrophage balance.

**Table 3:** The gene pathways in the induction of M2 macrophage polarization for TRDs

Gene pathways	Mediator	Effects for TRDs	References
NF-κB	miR-29a	Increase the Col I/Col III ratio and improve ECM remodeling	[47]
	ADSCs-EVs	Promote chondrogenic and osteogenic differentiation	[65]
	Healthy TDSCs derived Exos	Attenuate TDSCs senescence and improve tenogenic, chondrogenic, and osteogenic differentiation	[5]
	BG elicited MSC-EVs	Promote tenogenesis and angiogenesis	[31]
	Inflammation-primed ADSCs-EVs	Promote intrinsic healing of tendons	[143]
Nrf2-HO-1	Tannic acid	Eliminate ROS	[43]
	Melatonin	Reduce oxidant stress	[144]
MAPK	iMSC-lEVs	alleviate tendinopathy-related pain	[14]
	TDSCs derived Exos	Promote ECM remodeling	[145]
JAK-STAT	Parishin A	Inhibit chondrogenic and osteogenic differentiation to suppress heterotopic ossification	[146]
	Mechanical stimulation	Promote chondrogenic and osteogenic differentiation	[80]
PPAR	Pioglitazone	Promote the adipogenic differentiation of TDSCs	[147, 148]

*TRDs* tendon-related diseases, *Col* collagen, *ECM* extracellular matrix, *ADSCs* adipose-derived stem cells, *EVs* extracellular vesicles, *TDSCs* tendon-derived stem cells, *Exos* exosomes, *BG* bioactive glass, *MSC* mesenchymal stem cells, *ROS* reactive oxygen species, *iMSC-lEVs* large extracellular vesicles of induced pluripotent stem cell-derived mesenchymal stem cells, *NF-κB* nuclear factor-kappa B, *MAPK* mitogen-activated protein kinase, *JAK* Janus kinase, *STAT* signal transducer and activator of transcription, *PPAR* peroxisome proliferators-activated receptors, *Nrf2-HO-1* nuclear factor erythroid-2-related factor 2/heme oxygenase 1

#### Drug delivery

Oral medications often fail to achieve adequate local concentrations and frequently cause side effects, accelerating the development of targeted drug delivery. By delivering specific drugs to injury sites, macrophage-mediated inflammation can be downregulated, and pro-regenerative capabilities enhanced. Clodronate, the most well-studied macrophage-depleting drug, requires liposomal encapsulation due to its short half-life and low lipophilicity [149]. Clodronate’s metabolite irreversibly binds to ATP/adenosine diphosphate (ADP) translocase, disrupting the respiratory chain, causing mitochondrial dysfunction, and ultimately inducing macrophage apoptosis. Hays et al. [150] demonstrated that liposomal clodronate injections following anterior cruciate ligament reconstruction could improve the morphological and biomechanical properties of the TBI, reducing macrophage and TGF-β accumulation. While non-specific macrophage inhibition prevents granulation tissue formation, it also reduces early matrix deposition [151]. However, de la Durantaye et al. [152] observed enhanced tensile strength in repaired Achilles tendons, although clodronate-induced macrophage depletion reduced cell proliferation and ECM formation. The discrepancies between these studies may stem from factors such as tendon origin and macrophage depletion extent, as well as their specific effects on tendon tissues.

Traditional drug-delivery scaffolds often exhibit biphasic release behavior, which leads to high local drug concentrations and interferes with early tendon healing. Deng et al. [105] fabricated a bilayered membrane with sustained and unidirectional delivery of ibuprofen. The high-concentration side of the membrane could inhibit fibroblast proliferation and macrophage recruitment, while the low-concentration side, with aligned collagen nanofibers, promoted tendon healing. This unidirectional delivery regulates immune responses while avoiding drug-induced interference in tendon tissue, offering a new direction for future research. Excessive ROS production during the early stages of TRDs not only exacerbates oxidative stress and inflammation but also drives macrophages toward M1 differentiation [43, 153]. Tannic acid (TA) and melatonin, as antioxidant agents, suppress inflammation and excessive ROS production, thereby aiding tendon and TBI regeneration [43, 106]. Moreover, melatonin promotes the chondrogenic differentiation of bone marrow stromal cells (BMSCs) and cartilage matrix synthesis [106], offering promising potential for TBI treatment. Therefore, future research should focus on developing drug delivery systems that not only mitigate macrophage-mediated inflammation but also enhance tenogenic and chondrogenic expression.

#### Gene delivery

The delivery of inhibitor drugs to mitigate the M1-like phenotype often lacks specificity and may interact with off-target pathways, potentially causing undesirable side effects [154]. As a novel approach, precise gene delivery can silence target genes and protein production, overcoming these limitations. MicroRNAs (miRNAs), long non-coding RNAs (lncRNAs), and circular RNAs (circRNAs) are key regulators of macrophage polarization. Current evidence suggests that miR-29a inhibits M1 differentiation by attenuating inflammasome assembly, downregulating nuclear factor kappa-B (NF-κB) p65 expression, and blocking its nuclear translocation, which improves tendon fibrosis and ECM remodeling by inhibiting Col III synthesis [47]. Cyclooxygenase (COX), which synthesizes prostaglandins, plays a crucial role in post-injury tendon inflammation [155]. Ye et al. [155] reported that lncRNA COX-2 small interfering RNA (siRNA) significantly downregulates M1 markers [TNF-α, inducible nitric oxide synthase (iNOS), and IL-12] while upregulating M2 markers (Arg-1 and IL-10). Yang et al. [156] revealed that transfection of macrophages with COX siRNA nanoparticles in vitro resulted in a shift from M1 to M2 phenotype, enhancing tenocyte proliferation. Importantly, circRNA Cdyl could promote M1 polarization by blocking interferon regulatory factor 4 (IRF-4) nuclear translocation [157]. Among these, miRNAs are extensively studied due to their ability to bind multiple target genes synergistically [47].

However, chemically synthesized RNAs are unstable, prone to degradation, and may cause off-target effects [158]. To address these issues, using RNA editing tools such as the clustered regularly interspaced short palindromic repeats (CRISPR)-Cas13 system to eliminate pro-inflammatory cytokine production or promote M2 polarization represents a promising gene delivery approach. However, the effective delivery of the Cas13 system to macrophages is hindered by the immunogenicity of traditional viral vectors [159]. Accordingly, Wang et al. [62] encapsulated the Cas13 system within cationic nanoclusters functionalized with mannose, which is recognized by overexpressed mannose receptors on macrophages, and combined ROS-responsive materials to achieve on-demand release based on ROS levels. Nanoparticle-based gene delivery offers advantages such as targeted delivery and controlled release. However, future research should focus on improvements in immune compatibility and gene-loading efficiency and solve large-scale production issues despite complex manufacturing processes.

#### Exosome delivery

As previously mentioned, there are complex interactions between MSCs and macrophages. In vitro co-culture of BMSCs and macrophages was found to enhance M2 polarization, attributed to the paracrine exosomes of BMSCs [40]. Compared to MSCs, exosomes, a type of extracellular vehicle (EVs), offer several advantages. Firstly, exosomes/EVs have low immunogenicity due to their biomimetic membrane structure and low expression of major histocompatibility complex (MHC) molecules [142]. Secondly, they lack a complete genome and proliferative capacity, mainly transferring signaling molecules like miRNA and proteins, thus not causing direct cell malignant transformation [70]. Therefore, this section reviews the role of exosomes/EVs in modulating macrophages for the management of TRDs. Numerous studies have demonstrated the efficacy of exosomes and EVs derived from MSCs, as shown in Table 4 [14, 70, 143, 145, 160–167].

**Table 4:** The exosomes/EVs used to modulate macrophages in the healing of tendon related diseases

Exosomes/EVs	Strengths	Delivery scaffolds	Effects on macrophages	Applications	References
TDSCs-exosomes	Enhancing tropism and affinity for tenocytes, promoting BMSCs proliferation and expression	GelMA, Collagen@Polydopamine scaffold	Attenuating M1 and promoting M2 polarization, increasing IL-10 and reducing IL-6 expression	Rotator cuff tears, tendon to bone healing	[70, 145]
BMSCs-exosomes	Good Biocompatibility and stability, strong permeability	Direct joint injection, Chitosan/β-Glycerophosphate/Collagen Hydrogel	Inhibiting M1 and promoting M2 polarization via miR-23a-3p	Tendon-to-bone healing	[160, 161]
ADSCs-exosomes/EVs	Abundant availability, lower cost, preventing ectopic ossification	Collagen sheet, GelMA	Increasing M2 and inhibiting M1 surface marker expression, inhibiting M1 macrophages via miR-147-3p	Rotator cuff tendinopathy, Achilles tendon repair	[143, 162, 163]
iPSC derived-MSCs-lEVs	Enriching of DUSP2, DUSP3, and other Proteins	Direct injection	Repolarize macrophages from M1 to M2 phenotype via P38 MAPK signaling	Quadriceps tendinopathy	[14]
HUCMSCs-exosomes/EVs	Minimal immunogenicity, promoting chondrogenesis	Sodium alginate hydrogel	Inhibiting M1 and promoting M2 polarization	Tendinopathy	[164]
HUVECs-exosomes	Promoting TDSCs proliferation	Polychitosan hydrogel	Attenuating M1 and promoting M2 polarization	Achilles tendon injury	[165]
IPFP MSC-exosomes	Wide availability, lower cost	Sodium alginate hydrogel	Attenuating M1 and promoting M2 polarization	Tendon-to-bone healing	[166]
Dendritic cell-derived exosomes	Strong ability to promote tissue regeneration and regulate tendinopathy inflammation	Direct injection	Promoting M2 polarization	Tendinopathy	[167]

*EVs* extracellular vesicles, *TDSCs* tendon-derived stem cells, *BMSCs* bone marrow stromal cells, *IL* interleukin, *ADSCs* adipose-derived stem cells, *HUCMSCs* human umbilical cord mesenchymal stem cells, *HUVECs* human umbilical vein endothelial cells, *IPFP MSC* infrapatellar fat pad mesenchymal stem cells, *iPSC derived MSCs-lEVs* large extracellular vesicles secreted by induced pluripotent stem cell-derived mesenchymal stromal cells, *M1* pro-inflammatory macrophages*, M2* anti-inflammatory macrophages, *DUSP* dual-specificity phosphatase, *MAPK* mitogen-activated protein kinase

Exosomes and EVs are small membrane vesicles secreted by cells, sharing similar functions [37]. Importantly, they avoid unstable differentiation while preserving immunoregulation capability [145, 168]. He et al. [70] demonstrated that exosomes derived from TDSCs could attenuate LPS-induced M1 macrophages and enhance M2 macrophage distribution around TBI. Li et al. [160] revealed that BMSC-derived exosomes highly express miR-23a-3p, targeting IRF-1 and inhibiting M1 polarization. Biomaterials can influence macrophage-MSCs interaction by enhancing paracrine function [31], indicating that MSCs can be pretreated to promote the secretion of ideal exosomes. EVs from adipose-derived stem cells (ADSCs) pretreated with circadian rhythm enhance M1 macrophage inhibition, highlighting the importance of normal physiological conditions [169]. By inhibiting M1 macrophages, mitochondria-rich EVs derived from BMSCs have been shown to mitigate muscle degeneration in RCT [170]. Although the immunological effects of these exosomes can be attributed to their stemness, exosomes derived from non-MSCs also demonstrate similar outcomes. A recent study revealed that endothelial cell-derived exosomes could promote M2 polarization and TDSCs proliferation [165], indicating the prospects of non-MSC-derived exosomes in regulating macrophage polarization and promoting the repair of TRDs. Compared to gene delivery, exosome delivery offers greater feasibility and more straightforward implementation than gene editing [70]. Nevertheless, the delivery efficiency of exosomes requires further investigation. For instance, direct injection of exosomes is susceptible to shear stress damage during the injection process [15], and controlling their release when loaded onto hydrogel scaffolds remains an unresolved challenge. GelMA scaffolds form stable gels at rupture sites after photo-crosslinking, minimizing TDSCs-derived exosome loss and enabling gradual absorption by the body [145], thus emerging as promising delivery scaffolds.

### Enhancing stimulation approaches

#### Magnetic fields

When exposed to MFs, macrophages alter their intercellular processes associated with polarization [171]. MFs can influence the binding of integrin receptors on the macrophage membrane surface to arginine-glycine-aspartic acid (RGD), an adhesive protein embedded in the natural microenvironment [84, 171], contributing to the formation of focal adhesion. On the other hand, under the external MFs, magnetic nanoparticles can guide macrophage/MSCs targeting injured sites, enhancing cell enrichment at the target sites [172]. Indeed, the regulation of macrophage polarization depends on the type, intensity, and frequency of MFs. For example, MFs with a static component of 60 μT and an alternating component of 100 nT were found to stimulate the production of TNF-α and IFN-γ [173]. In contrast, pulsed electromagnetic fields and moderate to high-intensity MFs promote a pro-regenerative macrophage phenotype [172, 174]. Kang et al. [84] incorporated superparamagnetic iron oxide nanoparticles containing RGD ligands into a planar matrix and found that low-frequency oscillating MFs enhanced M2 polarization and macrophage adhesion, while high-frequency oscillating MFs promoted M1 polarization. The mechanisms of magnetic field-mediated immune cell regulation include modulating cell morphology, ion channel regulation [e.g., transient receptor potential melastain 2 (TRPM2)], and iron metabolism [172], suggesting that further mechanistic clarification requires multi-disciplinary cooperation.

#### Ultrasonic fields

Ultrasonic fields, particularly low-intensity pulsed ultrasound (LIPUS), have been shown to enhance the healing of bone fractures and TBI [29, 175]. LIPUS serves as a non-invasive and safe approach in the application of TE [81]. Li et al. [81] observed that the therapeutic effects of LIPUS were attenuated after macrophage depletion. Their subsequent study further confirmed that LIPUS could facilitate M1 macrophage accumulation and promote M2 polarization during the later stages of TBI healing [175]. The mechanisms underlying LIPUS-induced macrophage polarization have been shown to involve several signaling pathways, including the signal transducer and activator of transcription 1/signal transducer and activator of transcription 6/peroxisome proliferator-activated receptor γ (STAT1/STAT6/PPARγ) pathways [176], and Wnt2b/Axin/β-catenin pathways [177]. These findings provide valuable insights into the biological effects of LIPUS and potential applications in reducing inflammation in TRDs. Beyond its direct therapeutic effects, ultrasound-assisted delivery therapy has attracted significant interest given its ability to enhance the uptake efficiency of drugs and other macromolecules [178]. Ultrasound-mediated delivery can influence molecular endocytosis, thereby affecting macrophage polarization and the healing of TRDs.

#### Electrical stimulation (ES)

All cells maintain a membrane potential regulated by ion channels, which can be modulated not only by adjusting extracellular ion concentrations but also by controlling electric fields [179]. Macrophages regulate their biological behaviors, such as migration, polarization, and phagocytosis, by reorganizing the actin cytoskeleton in response to ES [180]. The parameters of ES influence the response of macrophages. In the study by Gu et al. [180], square-waveform ES activated the NF-κB signaling pathway, promoting M1 polarization, whereas sine-waveform ES promoted M2 polarization. They also found that polarization increased with ES intensity, but higher intensities were detrimental to polarization. While alternating current ES at appropriate frequencies benefits macrophage function, excessive intensity can cause cell damage or overactivation. Bianconi et al. [181] concluded that M0 and M1 macrophages upregulate M2 marker gene expression when exposed to direct current ES. Traditional ES methods involve non-electrode contact, limiting their ability to directly “educate” macrophages and often damaging surrounding tissues. Recent advances in ES approaches based on planar microelectrodes [182], or piezoelectric materials [183, 184], leveraging biomaterial properties, have provided new insights for precisely controlling macrophage polarization. Piezoelectric materials can convert the mechanical forces generated during tendon movement into electrical signals to modulate the immune microenvironment [184], thereby eliminating the problems associated with external electrode implantation or power sources.

**Fig. 4:**
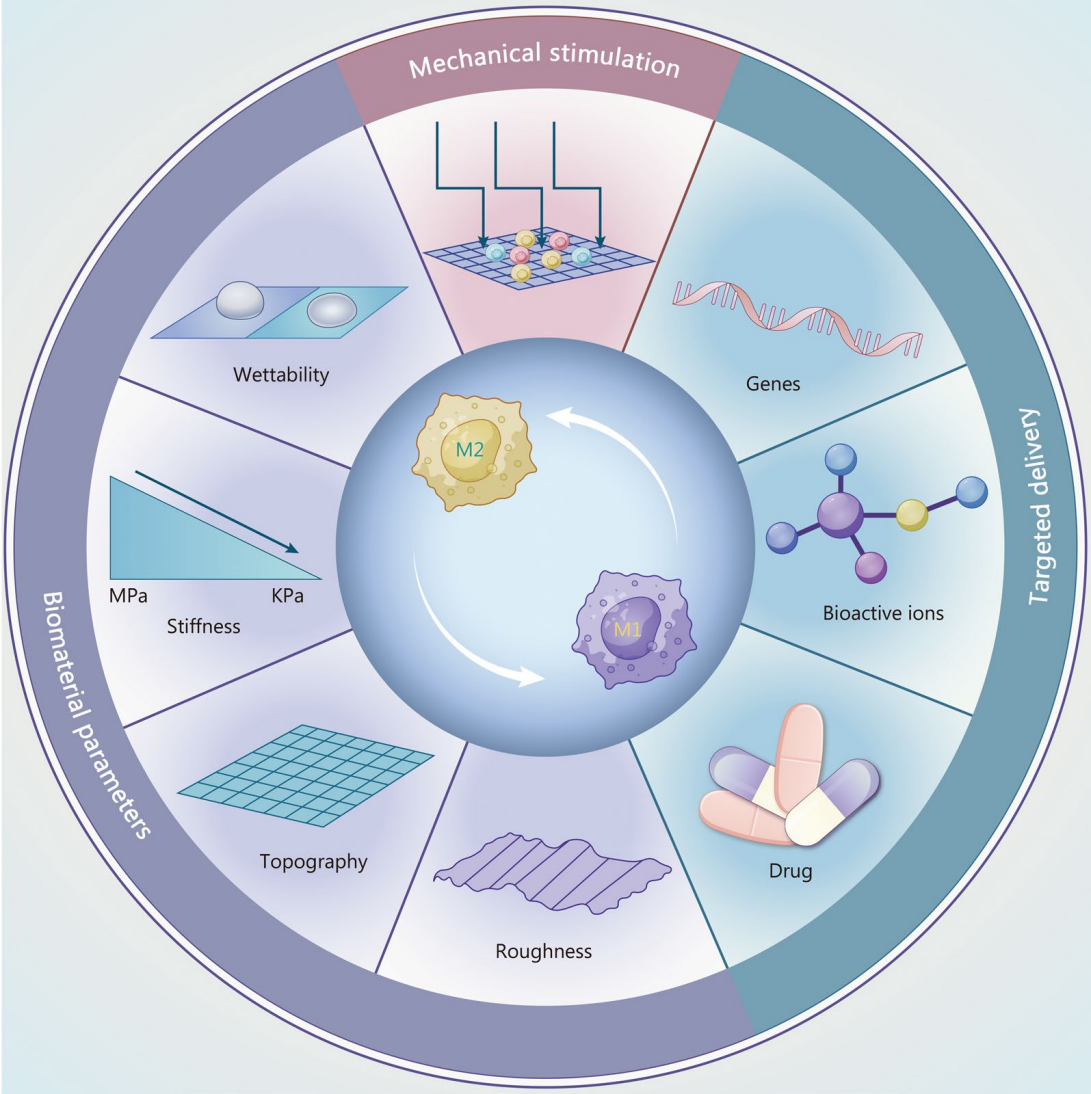
An overview of the strategies for macrophage polarization. The different parameters and stimuli can influence macrophage polarization towards either the M1 or M2 phenotype, including mechanical stimulation, targeted delivery (e.g., genes, bioactive ions, and drug), and biomaterial parameters (e.g., wettability, stiffness, roughness, and topography). M1 pro-inflammatory macrophages, M2 anti-inflammatory macrophages, MPa MegaPascal, KPa KiloPascal

Various methods exist for modulating macrophage polarization, and each has its advantages. Advanced nanomaterial fabrication techniques enable the creation of biomaterials with precise topography, stiffness, and other physical properties that can be loaded with bioactive factors, drugs, and genes. When combined with strategies for externally enhanced stimulation, these material-related parameters can synergistically influence macrophage gene expression and phenotype (Fig. 4**, **Table 5) [6, 47, 106, 109, 146, 156, 185–187]. However, regulating macrophage polarization should not depend exclusively on the combined effects of these methods. While synergistic regulation strategies are beneficial, researchers should prioritize approaches that enhance clinical translation and applications. Moreover, the long-term stability and effectiveness of composite biomaterials in regulating macrophage polarization need to be guaranteed. Given that some biomaterials may degrade or undergo property alterations over time, which can impact their regulatory effects on macrophage polarization, it is essential to integrate material degradation with the tendon-related disease repair cycle.

**Table 5:** The representative nanomaterials-based strategies in the regulation of immune response in the TRD healing

Nanomaterials	Cargos	Targets	Functions	References
Polymeric				
PLGA	COX siRNA	Achilles tendon injury	COX siRNA/PLGA nanoparticles can promote M2 polarization and the proliferation of tendon cells	[156]
PCL	bFGF	Achilles tendon defect model	PCL-bFGF scaffolds promote the formation of a tendon-like structure and M2 polarization	[185]
PCL	Melatonin	Rotator cuff tear	PCL nanofibers release melatonin sustainably which increases chondrogenic differentiation and inflammatory macrophage infiltration	[106]
Inorganic				
Mesoporous bioglass NPs	Sr	Torn rotator cuff	SrMBG can increase the number of M2 macrophages and the synchronous regeneration of the TBI composition	[6]
Mesoporous silica NPs (MSNs)	Parishin A	Tendinopathy	MSNs can release Parishin A sustainably, promote M2 polarization, and prevent heterotopic ossification	[146]
Hybrid				
GA-Ag NPs	-	Achilles tendon	GA-Ag NPs have anti-antibacterial, anti-adhesion, and anti-inflammation capabilities and promote M2 polarization	[109]
PELA/HA-LNPs	miR-29a	Achilles tendon	PELA/HA + miR29a-LNPs can induce the M2 polarization and accelerate tendon ECM remodeling	[47]
PLA/DLC	-	Achilles tendon adhesion model	PLA/DLC membrane can reduce the production of ROS and M1 polarization and the formation of adhesion	[186]
Lipid-PLGA NPs (LPNs)	Budesonide and Serpine1 siRNA	Tendinopathy	Dual-loaded LPNs promote macrophage polarization to M2 phenotype without affecting T cells and reduce fibrotic formation	[187]

*PLGA* poly lactic-co-glycolic acid, *PCL* polycaprolactone, *bFGF* basic fibroblast growth factor, *LNPs* lipid nanoparticles, *NPs* nanoparticles, *PELA* polylactic acid-Polyethylene glycol, *PLA* polylactic acid, *HA* hyaluronan, *miR29a* micro ribonucleic acid 29a, *DLC* diamond-like carbon, *ECM* extracellular matrix, *GA* gallic acid, *Ag* silver, *ROS* reactive oxygen species, *siRNA* small interfering RNA, *COX* cyclooxygenase, *M1* pro-inflammatory macrophages*, M2* anti-inflammatory macrophages*, MBG* mesoporous bioactive glass, *Sr* strontium, *TBI* tendon-bone interface

## Tissue engineering application of macrophage polarization for TRD treatment

### Tendon rupture

Acute tendon rupture initiates a natural healing cascade by recruiting immune cells and releasing pro-inflammatory cytokines, a process that can become prolonged and difficult to control. Excessive tendon inflammation dysregulates the coordinated collagen expression in fibroblasts and tenocytes, ultimately resulting in peritendinous adhesion. Besides, the inherent hypovascularity of tendons results in inadequate nutrient supply and vulnerability to mechanical stress from body movements. Macrophages, as pleiotropic immune cells, participate throughout the tendon healing process. Therefore, TE strategies focusing on immune system modulation have been explored to control inflammation and enhance tendon healing via the integration of immunoregulatory cells, factors, and customized scaffolds.

#### Application based on MSCs

TDSCs are progenitor cells that self-renew and differentiate into fibroblasts, maintaining tendon homeostasis [145]. TDSCs exhibit clonogenicity and self-renewal capacity, similar to ADSCs and BMSCs [188], contributing to the upregulation of tenogenic markers (Scx and Tnmd). Mao et al. [97] coated TDSCs onto the surface of small intestinal submucosa (SIS) to repair Achilles tendon defects, significantly enhancing tendon regeneration and inducing greater M2 polarization than TDSCs alone. Mechanically, SIS, a natural ECM scaffold, provides collagen fibers for tendon repair and a suitable microenvironment for TDSCs, while M2 polarization reduces adhesion formation. In the study by Gelberman et al. [189], combining BMP-12 with ADSCs sheets enhanced anti-inflammatory macrophage activation and increased MMP-12 production; While ADSCs alone regulate macrophage polarization, their effects are transient; BMP-12 prolongs this modulation by enhancing cell growth and tenogenic differentiation.

#### Application based on exosome/EVs

Despite the above benefits, MSC delivery poses risks such as immunogenicity and tumorigenic differentiation [161]. Moreover, stem cells primarily exert their effects through paracrine signaling via exosomes [40], driving the development of exosome-based modulation strategies. Exosome/EV-based macrophage modulation has been demonstrated to regulate macrophage immunophenotypes and accelerate tendon healing [168]. Macrophage-induced inflammation and insufficient tendon regeneration predominantly account for poor clinical outcomes following Achilles tendon rupture. Shen et al. [143] loaded inflammation-primed ADSCs-derived EVs into collagen sheets and observed increased tenocyte proliferation and collagen deposition at injury sites, inhibiting M1 macrophage activation simultaneously. Zhang et al. [145] loaded TDSCs-derived exosomes onto GelMA scaffolds to modulate post-rupture Achilles tendon inflammation, showing similar results with elevated IL-10 and tenogenic marker expression in the treatment group. Beyond balancing macrophage inflammation and promoting tenogenic expression, addressing the poor vascularity in injured tendons is another critical challenge. M2 macrophages enhance angiogenesis by promoting endothelial cell fusion and secreting pro-angiogenic factors. In this context, Xu et al. [31] reported a novel strategy to promote angiogenesis and tenogenesis. They used ADSCs-derived EVs primed by bioactive glass (BG), composed of bioactive ions (e.g., Si^4^⁺ and Ca^2^⁺). BG stimulation increased the production of specific miRNAs (miR-125a-5p and miR-199b-3p) that were associated with vascularization and immunoregulation. Notably, BG-stimulated EVs enhanced tenogenic differentiation without inducing heterotopic ossification [31]. Future studies should compare pretreatment strategies (with BG, bioactive agents, or pro-inflammatory factors) to enhance exosome/EV function and elucidate underlying mechanisms. Exosome/EV-mediated macrophage polarization involves multiple mechanisms, including miR-23a-3p [160], miR-147-3p [190], miR-125a-5p, and miR-199b-3p [31]. Nonetheless, the heterogeneous components of exosomes/EVs necessitate further exploration of specific signaling pathways.

#### Application based on self-healing biomaterials

The mechanical forces generated by tendon movement are often conducive to scaffold damage and detachment, with resulting fragments exacerbating immune responses and aggravating macrophage-mediated inflammation. Thus, endowing scaffolds with self-healing capabilities has become a research hotspot. Natural polymers such as chitosan, alginate, and hyaluronic acid have been widely explored to impart self-healing properties to bioactive scaffolds [191]. For instance, Wan et al. [192] designed an injectable hydrogel (CP@SiO_2_) comprising chitosan, hydroxyethyl cellulose, puerarin, and mesoporous silica nanoparticles. The CP@SiO_2_ hydrogel could promote TDSCs proliferation and M2 polarization, enhancing the mechanical properties of injured tendons. The glycoside Puerarin mitigated oxidative injury and inhibited inflammatory responses by self-assembling with chitosan in situ, improving the self-healing properties of the scaffold. The previously described BG-sodium alginate hydrogel with injectable and pro-angiogenic properties promoted the phenotypic switch of macrophages from M1 to M2 during Achilles tendon healing [12]. The injectable and self-healing properties of the hydrogel enabled complete defect filling, showing potential in minimally invasive orthopedic surgeries like arthroscopy. The chemically synthesized scaffolds possessing self-healing properties can revert to their original shape after being damaged, primarily through dynamic interactions (e.g., Schiff bonds and hydrazone bonds) [193]. In addition, numerous natural molecules (e.g., curcumin [194] and puerarin [192]) offer additional antioxidant and anti-inflammatory properties, making them ideal candidates for modulating macrophage balance in tendon repair.

#### Application based on scavenging ROS

Peritendinous adhesion is a significant complication following tendon rupture, arising from dysregulated extrinsic healing and collagen synthesis [195]. ROS accumulation after rupture creates a pro-inflammatory environment, raising the Col III/I ratio and leading to adhesion development and macrophage imbalance [196]. Significant efforts have been undertaken to leverage natural bioactive substances or traditional Chinese medicine to eliminate ROS and macrophage-mediated inflammation. As previously mentioned, TA attenuates inflammation and reduces oxidative stress due to its phenolic ligands. Zhao et al. [43] modified decellularized tendon scaffolds (DT) with TA to alleviate ROS-driven inflammation. They demonstrated that macrophages cultured on the modified scaffolds exhibited M2 polarization, with documented morphological changes and upregulated Arg-1 expression. Notably, DT scaffolds, with their specific natural tendon microenvironment and biocompatibility, effectively promote MSC proliferation and tendon differentiation, representing a novel strategy for reducing ROS. Consistently, gallic acid (GA) not only suppresses fibroblast adhesion but also exhibits antioxidant properties. Covalently grafting GA-silver nanoparticles (AgNPs) onto DT scaffolds reduced postoperative adhesion compared to DT scaffolds alone, by clearing ROS and promoting M2 macrophage polarization [109]. Moreover, GA’s antioxidant activity mitigated the cytotoxicity of AgNPs. Bioactive factors scavenge ROS through their antioxidant properties, and artificially synthesized nano-antioxidant enzymes have also shown significant effects. Rong et al. [29] developed a multifunctional enzymatic nanohybrid encapsulating EVs-BMSCs, combined with ultrasound stimulation to promote tendon matrix reconstruction and modulate the immune microenvironment. The nanohybrid not only scavenges ROS to promote M2 polarization but also suppresses Col III synthesis via Zn^2+^ release. Compared to natural enzymes, antioxidant enzymes offer significant promise due to their controllable catalytic activity and cost-effective production [60, 197]. Besides, biomaterial modification also presents a viable avenue for mitigating ROS production. In this respect, Xiao et al. [186] deposited diamond-like carbon (DLC) on the surface of polylactic acid (PLA) membranes to reduce tendon adhesion and immunological rejection, attributed to the abundant C=O groups of DLC that scavenged ROS production. Combining bioactive substances, nano-antioxidant enzymes, and biomaterial modification show potential in reducing peritendinous adhesion. ROS generation is closely linked to macrophage inflammation, suggesting that a synergistic strategy is needed to reduce the formation of peritendinous adhesions. Indeed, while adhesion formation is a long-term process, most experiments only assess short-term postoperative efficacy, neglecting the long-term stability of materials or nanozymes. This highlights the need for future experiments to further investigate these aspects.

#### Application based on genes

In addition, gene therapy represents a promising therapeutic approach. By delivering DNA or siRNA to target cells in the tissue, it can control the formation of peritendinous adhesions. The *Smad3* signaling plays a crucial role in adhesion formation in fibrotic diseases, and *Smad3* knockout has been demonstrated to suppress adhesive tissue formation. Cai et al. [193] encapsulated *Smad3*-siRNA in GelMA microspheres, which were degraded by MMP-2 overexpression during tendon healing. This innovative design facilitated the on-demand and unidirectional release of *Smad3*-siRNA nanoparticles, and incorporating it into the hyaluronan self-healing hydrogel prevented peritendinous adhesion significantly. However, their study [193] reported fewer M2 macrophages surrounding tissues with less adhesion, which seems to contradict the results of other studies. For instance, Chen et al. [47] concluded that miR-29a could inhibit the formation of peritendinous adhesions by improving M2 polarization and the immune microenvironment. They loaded miR-29a-lipid nanoparticles into the core layer of the PLA-polyethylene glycol (PEG) membrane to strengthen its anti-adhesive ability. Given that M2 macrophages play an essential role in inhibiting inflammasomes and improving the immune microenvironment, the differences in these research findings may be attributed to the varying immunogenicity of materials, such as polymeric materials, which are more capable of recruiting macrophages. However, research has indicated that M2 macrophages may also contribute to adhesion formation by secreting TGF-β1 [45], suggesting further investigation is needed for the M2 macrophages in the development of adhesion. Wang et al. [62] utilized CRISPR-Cas13 mRNA editing to suppress secreted phosphoprotein 1, an inflammatory protein expressed by macrophages during tendon injury, thereby reducing fibroblast activation and tendon adhesion. Gene regulation of macrophage polarization, particularly via gene-editing technologies, enables targeted cellular response regulation, offering a promising approach for improving tendon regeneration outcomes.

### TBI injury

#### Application based on relieving TBI inflammation

Following TBI injury, inflammatory factors released by macrophages accumulate at the injury site, leading to collagen disorganization, bone resorption, and impaired healing [67, 69]. Therefore, inhibiting inflammation is critical for addressing these challenges. Song et al. [106] fabricated aligned PCL electrospun membranes loaded with melatonin, which suppressed oxidative stress and inflammatory macrophages. The synergistic effect of aligned topography and melatonin promoted tenogenic and chondrogenic differentiation. Apoptotic cells accumulate post-injury, producing cytotoxic compounds and further exacerbating TBI-associated inflammation. Milk fat globulin protein E8 acts as an “eat me” signal, aiding macrophages in recognizing apoptotic cells [198]. Furthermore, macrophage efferocytosis enhances M2 macrophage polarization, creating a positive feedback loop for inflammation resolution [198].

MSC-derived exosomes/EVs also play a critical role in regulating TBI-associated inflammation. Huang et al. [67] treated rats with RCTs via intravenous injection of BMSC exosomes, inhibiting M1 macrophage-mediated inflammation and enhancing vascularization in the rotator cuff region. However, intravenous injection faces challenges in achieving sufficient exosome concentrations at the injury site. To prolong exosome release, Shi et al. [161] loaded exosomes into chitosan/β-glycerophosphate/collagen hydrogels, promoting M2 macrophage polarization and chondrogenic expression. These findings indicate that dysregulated TBI inflammation could be resolved synergistically through both polarizing macrophages and clearing apoptotic substances, based on composite strategies. Furthermore, the circadian rhythm mediated by melatonin facilitates MSC secretion of EVs that promote the differentiation of M2 macrophages. Song et al. [169] delivered EVs derived from circadian rhythm-pretreated ADSCs using a triphasic microneedle system for sustained release in TBI. The triphasic microneedle system consists of three components: tips for penetrating TBI tissue, a stem for sustained EV release, and a base incorporating decellularized tendon ECM to promote tenogenesis. This delivery system mitigated M1-mediated inflammation and facilitated fibrocartilage and tendon regeneration, offering a novel therapeutic strategy for TBI treatment. The microneedle system has the advantages of minimal invasiveness and thus reduced immune response [169]. By precisely delivering drugs or bioactive factors to modulate the local microenvironment, it shows great potential in regulating inflammation at the TBI.

#### Application based on accelerating synchronous regeneration

The naturally graded structure of TBI facilitates stress dissipation, prompting researchers to explore various strategies for synchronous regeneration across its distinct layers. Mesoporous BG incorporated with copper (Cu) has previously been shown to enhance TBI regeneration and modulate inflammation [107]. Gao et al. [6] recently incorporated mesoporous BG doped with strontium (Sr) into electrospun fiber scaffolds. The extract of the scaffolds upregulated osteogenic and chondrogenic markers in BMSCs (e.g., Runx2, Sox9, and Col2a1) and promoted M2 macrophage polarization, demonstrating dual-lineage induction and immunomodulatory effects. Given the dynamic nature of inflammatory responses during TBI healing, precise regulation of macrophages is essential. Li et al. [199] employed an innovative precision strategy for graded regulation of TBI-associated inflammation, comprising chemical regulators composed of PLGA-PEG-PLGA triblock copolymers and Mg^2+^-BMP-12 nanocomplex. Initially, free Mg^2+^ ions were released to promote M2 polarization, while sustained release from the nanocomposite established a pro-regenerative immune microenvironment, supporting synchronous regeneration of bone, fibrocartilage, and tendon. By binding with bioactive substances, the issue of metal ion burst release can also be addressed. Procyanidins not only scavenge free radicals but also chelate Mg^2+^ to achieve sustained release kinetics. Li and colleagues [200] incorporated Mg-procyanidin nanoparticles into a composite hydrogel composed of dopamine-modified hyaluronic acid and F127. The composite hydrogel underwent covalent crosslinking with Mg-procyanidins via catechol moieties, enabling sustained Mg^2+^ release for up to 56 days. Given the presence of a mineralization gradient at the TBI, the synchronous regeneration of its components is essential. The findings of these studies collectively demonstrate that multiphase metal ion scaffolds (Cu^2+^ and Sr^2+^) based on BG, or those enabling sustained release of metal ions, can induce synchronous regeneration at the TBI while modulating immune-inflammatory responses. Compared to gradient scaffolds based on cells or active factors, metal ions-containing gradient scaffolds offer two main advantages: 1) they can regulate macrophage responses and cellular enzyme synthesis, which helps promote the regeneration of cartilage and tendons [107]; 2) their concentration gradients are more comparable with the mineralization gradient at the TBI. This feature enables them to serve as a temporary patch during the initial stages of repair and support stress release. However, the concentrations of metal ions that promote the regeneration of different tissue layers require extensive experiments. Furthermore, the application of metal ion scaffolds necessitates careful consideration of biotoxicity thresholds.

In addition to multiphasic metal ion scaffolds, stem cell-integrated multicellular scaffolds provide an alternative solution to promote synchronous regeneration of the TBI. Du et al. [201] designed a multicellular scaffold with spatially distributed TDSCs and BMSCs. The manganese silicate nanoparticles in the scaffold performed two key functions: 1) they stimulated macrophages to secrete prostaglandin E2, which promoted BMSC osteogenic differentiation and TDSCs tenogenic differentiation within the scaffold via paracrine signaling; 2) they induced macrophage polarization toward an anti-inflammatory M2 phenotype, optimizing the regenerative microenvironment. While stem cell-based multicellular scaffolds show promise for TBI regeneration, further improvements are needed. For example, designing gradient-distributed biological scaffolds incorporating multiple stem cell-derived exosomes/EVs, traditional Chinese medicine, or bioactive factors could yield similar outcomes.

#### Application based on improving TBI comorbidities

Given that patients with TBI injuries, particularly RCTs, are predominantly middle-aged and older adults, managing osteoporosis is a critical consideration. Song et al. [65] fabricated a macroporous hydrogel (with pore sizes of hundreds of micrometers) composed of sodium alginate, hyaluronic acid, and ADSC-derived EVs using a wet spinning technique. The macroporous structure of the scaffold facilitates cellular infiltration and nutrient exchange, while its aligned topography promotes tenogenic differentiation of TDSCs. Furthermore, the EVs inhibited M1 polarization via the NF-κB pathway and upregulated BMP2 and Runx2 expression to enhance bone regeneration. Recent research has indicated that exosomes derived from M2 macrophages prevent other degenerative complications, such as muscle atrophy, fatty infiltration, and cellular senescence [11, 202]. Therefore, future research should focus on developing integrated therapeutic strategies that simultaneously promote M2 macrophage polarization and prevent these degenerative complications.

### Tendinopathy

Tendinopathy is pathologically characterized by disorganized collagen bundles, pathological neovascularity, and ectopic ossification [203]. Research has shown that the development of tendinopathy is associated with inflammation and dysregulation of the innate immune system, leading to chronic pathological progression. Fu et al. [204] classified tendinopathy into three developmental stages: the injury stage, the failed healing stage, and the clinical presentation stage. Tendon injury, often caused by overuse, triggers an inflammatory response that leads to collagen degradation. Prolonged repetitive mechanical loading causes sustained inflammation, undirected cell differentiation, heterotopic ossification, and pathological angiogenesis, making repair challenging. Chronic collagen and cellular metabolic disorders result in pain and spontaneous tendon rupture [204]. Therefore, modulating the immunological response to improve these pathological histological features through TE is a key solution.

#### Application based on MSCs or their derived exosome/EVs

ADSCs are commonly used in cell therapy for their easy availability and stemness. Kokubu et al. [162] treated collagenase-induced tendinopathy in mice via direct ADSCs injection, suppressing M1 polarization and the inflammatory response. The ADSCs also promoted early angiogenesis, reversing hypoxia and preventing ectopic ossification. ADSC-derived EVs can be categorized into exosomes and ectosomes based on their diameter, both of which are essential mediators of ADSC paracrine regulation [205]. ADSC-derived exosomes demonstrated therapeutic efficacy for tendinopathy when injected into lesion sites [163]. Xu et al. [205] reported that exosomes from ADSCs are more effective than ectosomes in treating Achilles tendinopathy. Notably, ADSC-derived ectosomes do not affect macrophage polarization, emphasizing the importance of characterizing their functions before applying MSC-derived EVs. Although the diameter of large EVs (> 200 nm) secreted by induced pluripotent stem cell-derived mesenchymal stromal cells (iMSC-lEVs) is different from the commonly extracted small EVs (< 200 nm), they showed similar therapeutic effects on quadriceps tendinopathy healing [14]. The iMSC-lEVs alleviated rat pain behavior by reducing inflammatory markers and repolarizing macrophages to an M2 phenotype via the p38 mitogen-activated protein K pathway [14]. Consequently, research on exosome therapy for inflammatory tendinopathy may not require the isolation of large exosomes in the future.

In the context of inflammatory tendinopathy, pro-inflammatory factors are synthesized in association with immune dysregulation. The antigen-presenting dendritic cells (DCs) and macrophages are frequently found in the peritendinous area. Their “cell chat” plays a significant role in tendinopathy development. In the Achilles tendinopathy model, injecting DC-derived exosomes regulated macrophage polarization via the phosphoinositide-3-kinase/Akt pathway promoted tenocyte differentiation, and inhibited type III collagen synthesis [167], demonstrating their potential for preventing tendinopathy. Future studies should combine DCs with the aforementioned biomaterial techniques to improve targetability and therapy for tendinopathy treatments.

#### Application based on sustained-release materials

Although the above studies have achieved some success in treating tendinopathy by regulating macrophage polarization using MSCs or their derived exosomes, several limitations remain. For instance, as previously mentioned, direct exosome injection can lead to rapid degradation, making it challenging to maintain a stable concentration and potentially causing a burst release. Li et al. [16] used DT scaffolds loaded with TDSCs-derived exosomes, achieving sustained exosome release for over two weeks. This combination enhanced M2 macrophage polarization, reduced inflammatory infiltration in tendinopathy, and increased the expression of tendon formation markers tenascin-C (TNC) and tenomodulin (TNMD). As a chronic condition, tendinopathy requires greater emphasis on using sustained-release platforms to regulate macrophage balance. Parishin A (PA), a traditional Chinese medicine, suppresses senescence-associated expression and molecular inflammation. Zhu et al. [146] loaded PA into mesoporous silica nanoparticles for sustained release, avoiding the need for repeated administration in cases of secondary injury. This combination enhanced macrophage CD206 expression and M2 polarization via the Janus kinase/STAT1 pathway, promoting tendon collagen alignment. Similarly, López et al. [187] developed dual-loaded nanoparticles containing budesonide for M2 polarization and Serpine1 siRNA targeting plasminogen activator inhibitor 1, a factor involved in fibrosis formation, to treat tendinopathy. The scaffold design employed a core–shell configuration, with budesonide incorporated in the core for sustained release and Serpine1 siRNA embedded in the lipid shell to enhance cell membrane penetration and promote targeted delivery. Thus, constructing a sustained-release platform requires integrating material properties with structural design.

#### Application based on addressing degenerative changes

Furthermore, as a degenerative disease, senescent cells and organelles should not be overlooked in tendinopathy healing. In this respect, current evidence suggests a positive feedback loop exists between senescent TDSCs and macrophages in aged rotator cuffs [5]. This positive feedback can be disrupted using healthy TDSCs-derived exosomes via BMP-4 signaling, reprogramming macrophages to an M2 phenotype. Degenerative mitochondria reduce ATP synthesis and increase ROS production, contributing to tissue injury and immune dysregulation [60]. Wang et al. [60] incorporated antioxidant cerium oxide nanozymes into a nanofiber scaffold (NBS@CeO) using dynamic liquid support electrospinning. This composite scaffold improved TDSCs metabolic function, promoted anti-inflammatory macrophages, and downregulated senescence, serving as a promising treatment strategy for degenerative tendinopathy. The aberrant neovascularization characteristic of tendinopathy contributes to structural tendon deterioration, making therapeutic angiogenesis inhibition a viable strategy for promoting tissue recovery. Li et al. [15] found that MSCs cultured in gelatin microcarriers exhibit favorable properties for suppressing vascularization in tendinopathy. Gelatin microcarriers formed tight cell connections and secreted matrix, creating microtissue constructs that effectively limited angiogenesis. Besides, mechanical stimulation from the microcarriers enhanced MSC paracrine function, inhibiting VEGF receptor expression in vascular endothelial cells. The role of macrophages in angiogenesis is complex, and few studies have explored treating tendinopathy based on their vascular regulatory effects. Future tissue-engineered constructs could leverage the angiogenic regulatory effects of different macrophage phenotypes to promote tendinopathy healing.

The modulation of macrophage polarization has emerged as a critical therapeutic strategy for repairing tendons, TBI regeneration, and tendinopathy management. Promoting the polarization of macrophages towards an anti-inflammatory phenotype is beneficial for the synchronous regeneration at TBI, preventing peritendinous adhesion, and ultimately alleviating patient pain to facilitate the recovery of TRDs. However, the dual nature of macrophages presents a significant challenge in regenerative therapies, and maximizing their beneficial role in TRDs is currently a major challenge. To leverage the regulation of macrophage polarization for tissue repair, a comprehensive approach is necessary by analyzing the specific roles of macrophages in the pathological changes of TRDs, fully integrating material parameters and bioactive regulators, and the application of physical stimuli to assist in regulation. This ensures that the phenotypic changes of macrophages focus on tissue regeneration rather than exacerbating tissue damage.

## Summary and future perspectives

As the trend of population aging intensifies, TRDs are becoming increasingly prevalent, significantly impacting citizens’ quality of life. The limited regenerative capacity of tendons and the TBI pose significant challenges for TRDs treatment. Modulating macrophage phenotypes to improve the regenerative microenvironment has demonstrated feasibility in managing various diseases. This review summarizes the impact of macrophages on TRDs development, strategies to promote macrophage polarization, and applications of macrophage polarization in TRDs healing. Macrophages exhibit a dual role in TRDs healing, encompassing both M1 and M2 phenotypes. M1 macrophages facilitate pathogen and cell debris clearance in the early post-injury phase, while M1-mediated inflammation also triggers peritendinous adhesion [206]. Although the M2 phenotype promotes tenogenic and chondrogenic differentiation [207], it can lead to fibrotic scar tissue formation [70]. Therefore, tendon or TBI regeneration requires precise modulation of macrophage phenotypes based on inflammatory levels to eliminate inflammatory factors or enhance tenogenesis and chondrogenesis. Strategies to modulate macrophage dysregulation are diverse and can be categorized into four main approaches: MS, biomaterial induction, targeted delivery, and physically enhanced stimulation. In tissue engineering for TRDs healing, these factors should be tailored to regulate macrophage reprogramming, as their behavior varies under different conditions. With rapid advancements in fabrication techniques, biomaterials are being designed with increasing precision, spanning micrometer to nanometer scales, 2D to 3D structures, and singular to composite regulation, providing precise modulation of macrophage behavior in TRDs treatment.

Currently, the role of macrophages in the healing of the TBI and tendons remains controversial. On one hand, the M2 phenotype creates a pro-healing microenvironment by secreting IL-4, IL-10, and TGF-β, which improves biomechanical strength through increased ECM accumulation [3, 208, 209]. On the other hand, it promotes the formation of fibrovascular and adhesive tissues rather than tendon-like tissues [32, 208]. Although M1 macrophages are traditionally considered pro-inflammatory, their depletion does not accelerate the healing process [151], highlighting the importance of an appropriate M1/M2 ratio. Besides, the underlying mechanisms of this phenomenon can be elucidated as follows: 1) the disparity between intrinsic and extrinsic healing processes within tendons. Tendons after acute injury exhibit a heightened extrinsic healing response that promotes fibrotic tissue expression (mediated by macrophages and fibroblasts), while intrinsic healing, which fosters tenogenic expression (mediated by tenocytes), is diminished; 2) macrophage heterogeneity, characterized by the further subdivision of M1 and M2 macrophages into subtypes such as M2a, M2b, and M2c. These subtypes possess distinct functions in immune regulation and possibly contribute to poor tendon or TBI healing [3]. Given that macrophage polarization represents a dynamic continuum of phenotypes, future research should focus on the characterization of macrophage subtypes on TRDs using multi-omics approaches. For example, single-cell genomics holds the potential to identify the specific subtypes of M2 macrophage responsible for vascularization and fibrosis at each stage of healing, thereby enabling the rational design of biomaterials with tailored parameters to induce favorable phenotypes for the healing of TRDs. Besides, the role of other immune cells, such as T cells and neutrophils, on the pathogenesis of TRDs remains unclear, though macrophages play a significant role within the immune system. The “cell chat” among these immune cells also warrants a comprehensive investigation to discover novel targets for the treatment of TRDs.

Strategies for modulating macrophage polarization should be based on the microenvironment of the tendon or TBI. For instance, beyond exhibiting low immunogenicity, biomaterials intended for TRDs treatment should possess comparable mechanical properties to withstand the forces generated during muscle contraction. To prevent immunological rejection caused by biomaterial fragmentation, self-healing properties are essential. Targeted delivery systems (e.g., exosomes, drugs, and genes) are necessary to regulate macrophage fate and enhance tenogenic, chondrogenic, and osteogenic expression. However, improving delivery efficiency, especially long-term efficiency, remains an unresolved problem, intricately linked to the design of biomaterials and enhanced stimulation methods, including electrical, ultrasound, and magnetic field stimulation. Nano-packaging materials and responsive controlled-release systems hold promise in these fields. While strategies for macrophage regulation can be categorized into the aforementioned protocols, they are not mutually exclusive, and a promising TE product should integrate them to maximize therapeutic effects. In the era of artificial intelligence (AI) and big data, previous experimental data can be compiled to establish a learning database, enabling AI to select optimal parameters for biomaterial-based delivery design, thereby creating ideal therapeutic products for TRDs. Last but not least, although current tissue engineering by macrophage regulation has achieved promising results in treating TRDs experimentally, its clinical applicability remains to be determined. In this respect, complicated preparation and high costs hinder its promotion. Therefore, future researchers need to prioritize clinical translation, improve biomaterials, simplify preparation, and enhance repair effects and practicality.

In conclusion, TRDs treatment is a complex process influenced not only by the intricate structure of tendons but also by their pathogenesis. Macrophages play a crucial role in these processes, and their functional plasticity can be modulated by TE to promote the healing of TRDs. TE approaches targeting macrophage imbalance hold promise for the treatment of TRDs and serve as potential therapeutic methods in future clinical settings.

## Data Availability

Not applicable.

## Abbreviations

2D: Two-dimensional
3D: Three-dimensional
ADSCs: Adipose-derived stem cells
Arg-1: Arginase-1
ATP: Adenosine triphosphate
BMSCs: Bone marrow stromal cells
BMP: Bone morphogenetic protein
BG: Bioactive glass
CD: Cluster of differentiation
Col I: Type I collagen
Col III: Type III collagen
COX: Cyclooxygenase
DC: Dendritic cell
DT: Decellularized tendon
ECM: Extracellular matrix
EVs: Extracellular vesicles
GA: Gallic acid
IL: Interleukin
IFN-γ: Interferon-γ
LIPUS: Low-intensity pulsed ultrasound
LPS: Lipopolysaccharide
MFs: Magnetic fields;
miRNAs: Micro ribonucleic acids
MMP: Metalloproteases
MS: Mechanical stimulation
MSCs: Mesenchymal stem cells
PCL: Polycaprolactone
RCTs: Rotator cuff tears
ROS: Reactive oxygen species
SIS: Small intestinal submucosa
siRNA: Small interfering RNA
TA: Tannic acid
TBI: Tendon-bone interface
TDSCs: Tendon-derived stem cells
TE: Tissue engineering
TGF-β: Transforming growth factor-β
TNF-α: Tumor necrosis factor-α
TLR: Toll-like receptor
TRDs: Tendon related diseases
TNC: Tenascin-C
TNMD: Tenomodulin
VEGF: Vascular endothelial growth factor
